# Factors linked to informal caregiver burden in dementia across Latin America and the Caribbean: A systematic review and meta‐analysis

**DOI:** 10.1002/alz.71506

**Published:** 2026-06-01

**Authors:** Paula Quintero‐Cardona, María Alejandra Tangarife, John Blandon, Juan Carlos Arango‐Lasprilla, Agustin Ibanez, Sandra Baez

**Affiliations:** ^1^ Universidad de los Andes Bogotá Colombia; ^2^ Universidad Europea del Atlántico Santander España; ^3^ IGC Pharma SAS Bogotá Colombia; ^4^ Social Research Institute, Institute of Education University College London London UK; ^5^ Department of Cell Biology and Histology University of the Basque Country UPV/EHU Leioa Spain; ^6^ IKERBASQUE Basque Foundation for Science Bilbao Spain; ^7^ Barcelonaβeta Brain Research Center (BBRC) Pasqual Maragall Foundation Barcelona Spain; ^8^ Cognitive Neuroscience Center Universidad de San Andrés Buenos Aires Argentina; ^9^ Latin American Brain Health Institute (BrainLat) Universidad Adolfo Ibañez Santiago de Chile Chile; ^10^ Department of Biophysics, School of Medicine Istanbul Medipol University Istanbul; ^11^ Global Brain Health Institute (GBHI) Trinity College Dublin Dublin, Dublin Ireland

**Keywords:** caregiver burden, cognition, Dementia, depression, education, functional dependence, gender, informal care, Latin America, meta‐analysis, neuropsychiatric symptoms, quality of life, systematic review

## Abstract

Informal caregivers are central to dementia care in Latin America and the Caribbean (LAC), yet determinants of caregiver burden remain insufficiently characterized. We conducted a systematic review and meta‐analysis of studies examining patient‐ and caregiver‐related correlates of burden in LAC. Random‐effects models pooled Fisher's z‐transformed correlations. Forty studies were included, of which 34 (*n* = 3,082 caregivers) were meta‐analyzed. Higher burden was associated with the patient's neuropsychiatric and depressive symptoms, and caregiver depressive and anxiety symptoms. Better patient cognition and quality of life were associated with lower burden. Meta‐regressions showed that higher caregiver education strengthened the association between patient depressive symptoms and burden, and a higher proportion of female caregivers strengthened the association between patient cognition and burden. Findings highlight the role of behavioral and affective symptoms and support culturally grounded, gender‐sensitive strategies in LAC. High heterogeneity underscores the need for methodological harmonization and further regional research.

## Highlights

- First meta‐analysis of informal caregiver burden in dementia across Latin America and the Caribbean (LAC).
- Caregiver depression and patient neuropsychiatric symptoms showed the strongest associations.
- Better patient quality of life and cognition were associated with lower caregiver burden.
- Higher education reduced, and female gender intensified, caregiver burden.
- Findings support culturally grounded and gender‐sensitive dementia care strategies.

## 1 BACKGROUND

Dementia caregiving in Latin America and the Caribbean (LAC) occurs within a regional epidemiological and socio‐structural context marked by rapid demographic aging, high dementia prevalence, and persistent structural inequalities.^1^, ^2^, ^3^, ^4^ Prevalence rates are among the highest globally and are projected to rise substantially in coming decades.^5^ Across the region, care provision relies predominantly on unpaid informal family caregivers, as public long‐term care systems remain underdeveloped and unevenly implemented.^1^, ^3^, ^6^ Although LAC countries differ economically and politically, they share structural challenges in dementia care, including fragmented policy implementation and persistent gendered caregiving expectations.^1^, ^3^, ^7^ Consequently, dementia care is largely sustained by families operating within constrained health and social protection systems.

In contrast to many high‐income countries, where long‐term care systems partially redistribute caregiving responsibilities, caregiving in LAC remains largely concentrated within families and supported by limited formal services.^1^, ^6^, ^8^, ^9^ Cultural norms centered on filial obligation and familism further reinforce this expectation, often discouraging help‐seeking behaviors.^10^, ^11^ Consequently, caregivers frequently experience substantial burden, reflected in emotional strain, physical exhaustion, social isolation, financial strain, and adverse mental health outcomes.^5^, ^11^, ^12^, ^13^ These shared structural characteristics across LAC underscore the need for regionally grounded evidence to inform strategies aimed at addressing caregiver burden.

A growing body of research has examined sociodemographic, psychological and clinical factors associated with caregiver burden in LAC.^14^, ^15^, ^16^ However, this evidence remains fragmented, with substantial variability in study design, sample characteristics, and measurement approaches, and a tendency to examine patient‐ and caregiver‐related factors in isolation. Previous meta‐analyses^17^, ^18^, ^19^, ^20^, ^21^, ^22^, ^23^, ^24^ have largely focused on high‐income settings, isolated predictors, or mixed clinical populations, limiting their relevance to LAC. To date, no meta‐analysis has quantitatively synthesized the simultaneous contribution of multiple determinants of caregiver burden within this region. By estimating the relative strength of these correlates within a single regional framework, the present study contributes context‐specific evidence that may help inform context‐specific research, intervention design, and policy development in LAC.

Conceptually, the integration of multiple determinants in this study is guided by the Stress Process Model,^25^, ^26^ which conceptualizes caregiver burden as the result of exposure to primary stressors embedded in caregiving demands and shaped by caregivers’ background characteristics. Within this framework, patient‐related factors such as neuropsychiatric symptoms, depressive symptoms, cognitive decline, and functional impairment operate as core primary stressors embedded in caregiving demands. Caregiver characteristics including education, age, and sex represent contextual conditions that may influence vulnerability to these stressors. This model provides a coherent structure for examining how multiple patient‐ and caregiver‐related factors jointly contribute to caregiver burden in LAC.

Against this background, the present study undertakes a systematic review and meta‐analysis of factors associated with caregiver burden among informal caregivers of people living with dementia in LAC. This review synthesizes evidence across major dementia subtypes, including Alzheimer's disease (AD), vascular dementia, frontotemporal dementia, and Lewy body dementia, examining both caregiver‐related factors (e.g., depressive and anxiety symptoms, education, sex, and age) and patient‐related clinical factors (e.g., neuropsychiatric symptoms, depression, cognitive status, functional ability, quality of life, diagnosis, and disease awareness). By integrating these determinants within a regional analysis, this study provides context‐specific evidence to inform research, intervention design, and policy development in LAC.

## 2 METHODS

**FIGURE 1 alz71506-fig-0001:**
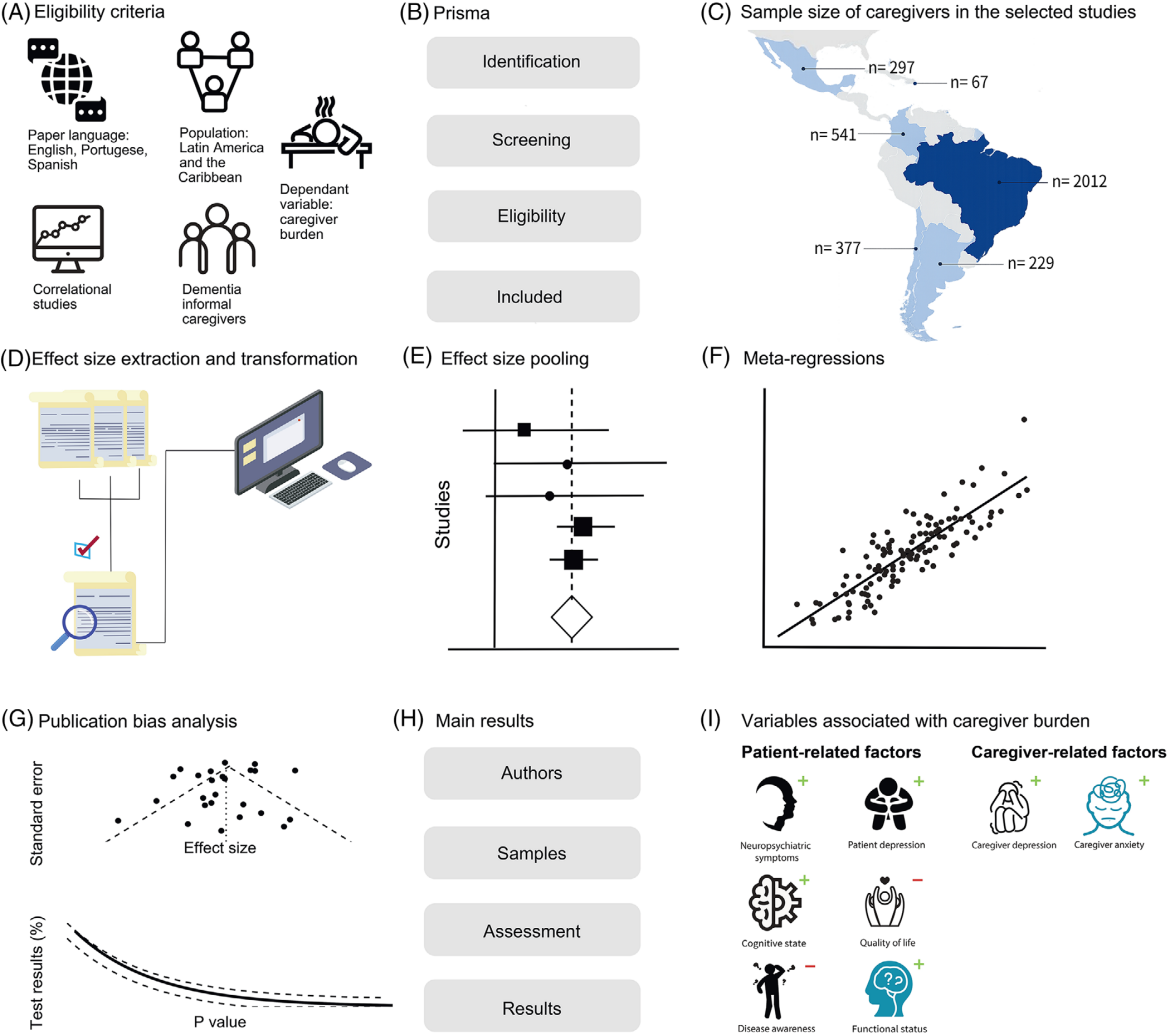
Flow diagram and analytic steps of the systematic review and meta‐analysis. (A) Eligibility criteria: informal caregivers of individuals with dementia in LAC; quantitative studies reporting associations with caregiver burden; publications in English, Spanish, or Portuguese. (B) Study identification: database searches conducted in MEDLINE via Ovid, PubMed, PsycINFO via APA PsycNet, and Web of Science Core Collection, complemented by manual screening of reference lists. (C) Geographic distribution: map of LAC indicating countries represented in the included studies and corresponding caregiver sample sizes. (D) Data extraction and transformation: extraction of effect sizes, standard errors, and *p*‐values; correlation coefficients were transformed to Fisher's z scores. (E) Meta‐analytic approach: pooling of effect sizes using random‐effects models; forest plots used to display individual and pooled estimates. (F) Meta‐regression analyses: examination of caregiver‐ and patient‐related moderators, including age, sex, education, and diagnosis. (G) Publication bias assessment: evaluation using funnel plots and Egger's regression test. (H) Summary of study characteristics: overview of included study features and key variables. (I) Factors associated with caregiver burden: schematic representation of patient‐ and caregiver‐related correlates included in the meta‐analysis; positive and negative associations are indicated with “+” and “–”, respectively. LAC, Latin America and the Caribbean.

This systematic review and meta‐analysis were conducted in accordance with the Preferred Reporting Items for Systematic Reviews and Meta‐Analyses (PRISMA) 2020 guidelines.^21^ The protocol was registered in the PROSPERO database (International Prospective Register of Systematic Reviews; ID: CRD420251043007). Figure 1 summarizes the methodological process of this study.

**RESEARCH IN CONTEXT**

1. **Systematic review**: We conducted a systematic review and meta‐analysis following Preferred Reporting Items for Systematic reviews and Meta‐Analyses (PRISMA) 2020 guidelines (PROSPERO CRD420251043007) across four databases (MEDLINE (via Ovid), PubMed, PsycINFO (via APA PsycNet), and Web of Science Core Collection). Forty studies from LAC examining correlates of caregiver burden in dementia were identified and critically appraised, of which thirty‐four met criteria for meta‐analysis.
2. **Interpretation**: Neuropsychiatric and depressive symptoms in individuals with dementia and caregiver depression showed the strongest positive associations with burden, whereas better patient cognition and quality of life were protective. These findings integrate previously fragmented regional evidence and reveal key psychological, social, and structural mechanisms underlying caregiver strain in LAC.
3. **Future directions**: Future research should apply longitudinal and mixed method designs to clarify causal pathways between patient and caregiver factors, include underrepresented LAC regions, and develop and validate culturally adapted measures of burden. Strengthening regional collaborations and research networks will be essential to translate this evidence into equitable caregiver support strategies and dementia care policies.

### 2.1 Eligibility criteria and information sources

Peer‐reviewed quantitative studies conducted in LAC that examined factors associated with caregiver burden among informal caregivers of individuals diagnosed with dementia were included. Eligible studies: (i) were published in English, Spanish, or Portuguese; (ii) reported correlational or regression analyses using caregiver burden as the dependent variable; (iii) examined at least one psychosocial or patient‐related factor (e.g., caregiver sex, education, depression; patient diagnosis, cognition, functionality, or neuropsychiatric symptoms); (iv) focused on informal caregivers, defined as family members or close relatives without formal training or financial compensation; (v) included caregivers of individuals clinically diagnosed with dementia, either unspecified or classified as AD, vascular dementia, frontotemporal dementia, Lewy body dementia, Parkinson's disease dementia, or mixed dementia, whether or not patients were directly assessed within the same study; studies including Parkinson's disease without dementia were excluded; and (vi) included caregivers aged 18 years or older.

Studies that: (i) focused on formal caregivers; (ii) included caregivers of individuals with diagnoses other than those specified above; (iii) used qualitative methods, were systematic reviews or meta‐analyses, or were conference proceedings, posters, or oral communications; (iv) were conducted outside the LAC region; or (v) included caregivers under the age of 18 were excluded. Only peer‐reviewed journal articles were considered eligible.

### 2.2 Search strategy

Four electronic databases, MEDLINE (via Ovid), PubMed, PsycINFO (via APA PsycNet), and Web of Science Core Collection, were systematically searched from inception to February 2026, with no restrictions on publication year. The search combined the terms “caregivers,” “burden,” and “dementia” with geographic keywords (e.g., “Latin America,” “Caribbean,” “América Latina,” as well as the names of individual LAC countries) in English, Spanish, and Portuguese. In addition, reference lists of all included studies and relevant reviews were manually screened to identify further eligible articles. The selected databases were chosen to ensure comprehensive coverage of biomedical, psychological, and multidisciplinary literature relevant to dementia caregiving in LAC

### 2.3 Study selection

**FIGURE 2:**
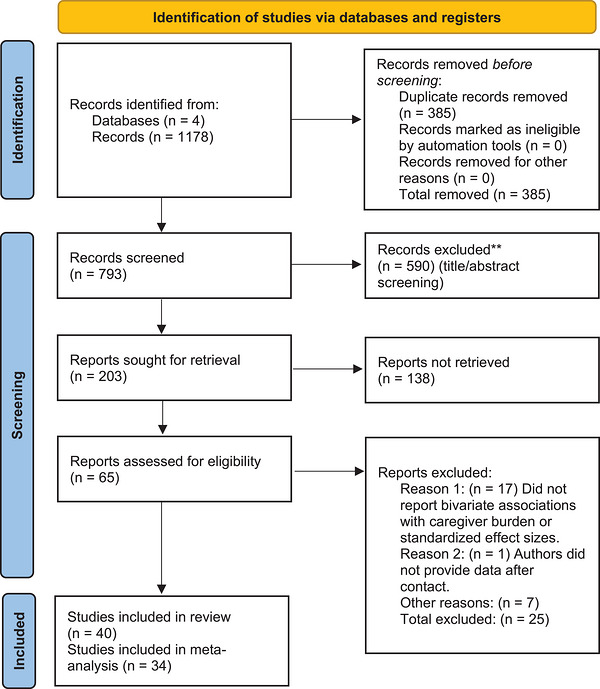
PRISMA 2020 flow diagram of the study selection process. The database search identified 1,178 records across four databases (MEDLINE, PubMed, PsycINFO, and Web of Science). After removing 385 duplicates, 793 records were screened by title and abstract, of which 590 were excluded. A total of 203 reports were sought for retrieval, and 65 full‐text reports were assessed for eligibility. Following full‐text assessment, 40 studies met inclusion criteria for the systematic review. Of these, 34 studies provided sufficient data to be included in the quantitative synthesis (meta‐analysis). Reasons for exclusion at the full‐text stage included lack of bivariate associations with caregiver burden or standardized effect sizes (*n* = 17), data not provided after author contact (*n* = 1), and other specified reasons (*n* = 7). PRISMA, Preferred Reporting Items for Systematic Reviews and Meta‐Analyses.

The study selection process is illustrated[Fig alz71506-fig-0001] in Figure 2. Three reviewers (P.Q.C., M.A.T., and V.A.) independently screened all records retrieved from the search. Duplicates were removed, and titles and abstracts were evaluated to identify potentially eligible studies. Full‐text articles were then assessed against the predefined inclusion and exclusion criteria. Disagreements during any stage of screening were resolved through discussion or consultation with two additional reviewers (J.B. and S.B.). All screening procedures were conducted manually and independently, and no automation or machine learning tools were used.

### 2.4 Data collection process and data items

Two reviewers (P.Q.C. and M.A.T.) independently extracted data from each included study using a standardized form. Extracted information comprised: (i) study characteristics (e.g., sample size, country), (ii) participant demographics (mean age, sex distribution, education of patients and caregivers), (iii) caregiving context (duration and diagnosis of the care recipient), and (iv) methodological details (instruments used to assess caregiver burden and associated psychosocial or patient‐related variables). When essential information was unavailable, study authors were contacted up to three times to request clarification or missing data. Any discrepancies between reviewers were resolved by consensus or, when needed, consultation with a third reviewer (S.B.). When key data (e.g., caregiver education) remained unavailable, the study was retained for qualitative synthesis but excluded from quantitative analyses such as meta‐regressions.

### 2.5 Outcome

The primary outcome across studies was caregiver burden. Variables associated with caregiver burden were not predefined; instead, they were identified during data extraction from the included studies and grouped according to conceptual similarity. Because this review aimed to quantitatively synthesize associations reported in the existing literature, all conceptually compatible measures of the same construct were considered. Only variables examined in at least five studies were eligible for meta‐analysis. Most studies assessed burden with the Zarit Burden Interview (ZBI), while a minority employed other conceptually similar measures, such as the Screen for Caregiver Burden (SCB) or the Caregiver Distress Index (CDI). Caregiver‐related variables included depressive symptoms, anxiety, quality of life, well‐being, and coping‐related psychosocial factors (e.g., sense of mastery). Caregiver age, sex, and education were tested as moderators in the meta‐regression analyses. Patient‐related variables included neuropsychiatric symptoms, cognition, functional status, disease awareness, and patient quality of life.

### 2.6 Risk of bias assessment

Risk of bias was assessed using the Newcastle‐Ottawa Scale (NOS)^27^ for cross‐sectional studies. Two reviewers independently (P.Q.C. and M.A.T.) rated each study across three domains: participant selection, group comparability, and outcome assessment. Discrepancies were resolved by consensus. Studies were scored from 0 to 9 (Scores: ≥7, high quality; 5–6, moderate; <5, low quality).^27^, ^28^

### 2.7 Synthesis methods

Given that the primary aim of this study was to quantify the strength and direction of associations between caregiver burden and related variables, correlational effect sizes were selected as the common metric for synthesis. Pooling correlation coefficients allowed comparability across studies using different instruments while preserving the magnitude of associations. Correlation coefficients were converted to Fisher's z scores to normalize distributions before pooling. A random‐effects framework was adopted a priori given the anticipated clinical, cultural, and methodological heterogeneity across countries, dementia subtypes, and measurement instruments within the LAC region. Effect sizes were pooled using restricted maximum likelihood (REML) estimation, which provides robust variance estimates under between‐study variability.^29^ Following recommended standards,^29^ only variables reported in five or more independent studies were included in the pooled analyses to ensure adequate statistical power and estimate stability.

When multiple instruments were available for the same construct, the validated measure most widely used in the literature was prioritized (e.g., ZBI for caregiver burden). When multiple effect sizes for the same association were reported within a study, a single effect size was selected based on conceptual relevance and consistency with the primary construct assessed, prioritizing the most commonly used and validated measure across studies.

Normality of correlation distributions was tested using the Shapiro–Wilk test, and Spearman's rho was used for non‐normally distributed variables. Effect sizes were interpreted as small (*r* = 0.10), moderate (*r* = 0.30), and large (*r* = 0.50) according to Cohen's conventions. Between‐study heterogeneity was assessed using I^2^ (with 50% considered moderate) and Cochran's Q statistic (*p* > 0.05 indicating no significant heterogeneity).^30^, ^31^ Influence analyses were performed with a leave‐one‐out approach to identify outliers and test the robustness of pooled estimates.^32^ Forest plots were generated to visualize individual and pooled effect sizes.

Meta‐regressions were conducted to examine whether caregiver age, sex, education, and patient diagnosis moderated the associations between caregiver burden and the study variables. Patient diagnosis was coded at the study level according to how the clinical sample was described in each study: (1) AD, including late‐ and young‐onset AD, and (2) mixed samples including AD and other dementia types (including frontotemporal dementia, Parkinson's, vascular, Lewy body, mixed, or unspecified types). Because most primary studies did not report results separately by subtype, this variable was treated as a pragmatic study‐level descriptor rather than a strictly mutually exclusive diagnostic classification. For neuropsychiatric symptoms, the total score of the Neuropsychiatric Inventory‐12 (NPI‐12) was used in the primary meta‐analyses, as it was the most consistently reported and validated measure across studies. When available, individual NPI symptom domains (e.g., agitation, apathy, hallucinations, irritability) were examined in separate exploratory analyses based on five independent studies (*k* = 5), and these results are reported independently. Influence analyses were conducted for all regression coefficients to assess whether any individual study disproportionately affected the moderation between caregiver burden and the selected outcome measures. All analyses were conducted in RStudio (version 2024.12.0) using the metafor package.^33^

### 2.8 Reporting bias assessment

We assessed reporting bias using both visual and statistical approaches. Funnel plots were generated to examine the symmetry of effect sizes in relation to study precision. Egger's regression test was applied to statistically evaluate small‐study effects, testing whether the magnitude of the effect size was associated with its standard error.^31^ A *p*‐value below 0.10 was considered indicative of potential publication bias, as recommended for meta‐analyses involving fewer than 10 studies per comparison^34^, ^35^.

## 3 RESULTS

### 3.1 Study selection

The database search yielded 1,178 records (MEDLINE via Ovid = 59, PubMed = 402, PsycINFO via APA PsycNet = 322, Web of Science = 395). After removing 385 duplicates, 793 unique records were screened by title and abstract. Of these, 203 reports were sought for retrieval, and 65 full‐text reports were assessed for eligibility. Following full‐text assessment, 40 studies met inclusion criteria^14^, ^15^, ^16^, ^36^, ^37^, ^38^, ^39^, ^40^, ^41^, ^42^, ^43^, ^44^, ^45^, ^46^, ^47^, ^48^, ^49^, ^50^, ^51^, ^52^, ^53^, ^54^, ^55^, ^56^, ^57^, ^58^, ^59^, ^60^, ^61^, ^62^, ^63^, ^64^, ^65^, ^66^, ^67^, ^68^, ^69^, ^70^ of which 34^15^, ^16^, ^36^, ^37^, ^38^, ^40^, ^41^, ^43^, ^44^, ^45^, ^46^, ^47^, ^48^, ^49^, ^50^, ^51^, ^52^, ^53^, ^55^, ^56^, ^57^, ^59^, ^60^, ^61^, ^62^, ^63^, ^64^, ^65^, ^66^, ^67^, ^68^, ^69^, ^70^, ^71^ were included in the quantitative synthesis (Figure 2). The remaining six studies^14^, ^39^, ^42^, ^54^, ^63^, ^72^ were excluded from the meta‐analysis because the variables they examined in relation to caregiver burden were reported in an insufficient number of studies to permit quantitative synthesis.

The systematic review included 40 studies across six LAC countries, mostly cross‐sectional and conducted in Brazil (Table S2). Caregivers were predominantly female (80.2%) and had a weighted mean of 10.53 years of education. The pooled sample comprised 3,523 participants, including 2,425 caregiver–patient dyads and 1,098 caregivers assessed independently. Studies not included in the meta‐analysis showed considerable heterogeneity in design and measurement instruments. Among variables that did not reach the threshold for quantitative pooling, satisfaction with life was assessed in four studies and showed negative associations with caregiver burden.^15^, ^58^, ^68^, ^69^ Caregiver education was analyzed in four studies^16^, ^36^, ^46^, ^57^ and was predominantly negatively associated with burden. Subscales of the NPI‐12, including disinhibition and nighttime disturbances were each examined in four studies and showed consistently positive correlations with caregiver burden.^16^, ^36^, ^49^, ^66^ Dementia severity was evaluated in four studies^36^, ^46^, ^48^, ^55^ showing positive correlations, with greater severity linked to higher burden. Caregiver health‐related quality of life was assessed in two studies using the 36‐Item Short Form Health Survey (SF‐36)^15^, ^58^ and showed negative associations with burden. Other variables examined only in isolated studies included patient physical activity,^45^ caregiver mastery,^42^ competence^37^, and social skills,^14^ which were generally associated with lower caregiver burden, except for competence, which showed a positive association.

Only variables reported in at least five studies were eligible for the meta‐analysis.^29^ A total of 34 studies (*k* = 34) met this criterion, including 3,082 caregivers (2,324 paired with patients and 758 assessed independently). Across studies, caregivers were predominantly women (80.1%) with a mean age of 56.94 years (SD = 3.11) and an average of 10.89 years of education (SD = 3.05). Patients had a mean age of 75.19 years (SD = 6.41). Twelve studies did not report patient age, one omitted caregiver age, and eight lacked data on caregiver education. In all studies, patients had a clinical diagnosis of dementia, most commonly AD, followed by vascular, frontotemporal, and mixed dementias. Three studies also included small subsamples of individuals with Parkinson's disease dementia (Perrin et al., 2014; Sutter et al., 2014; *n* = 2). In addition, Rosas‐Carrasco et al. (2014) reported a combined subgroup of frontotemporal, Lewy body, and Parkinson's disease dementias (*n* = 14), without disaggregating the number of Parkinson's disease dementia cases. The included studies were published between 1993 and 2025 and were conducted primarily in Brazil (61.76% – 21 studies), followed by Colombia (17.65% – 6 studies), Argentina (8.82% – 3 studies), Chile (2.94% – 1 study), Mexico (2.94% – 1 study), and the Dominican Republic (2.94% – 1 study), with one additional multi‐country study (2.94%) including participants from Argentina and Mexico. Sample sizes ranged from 25 to 291 participants.

Measurement tools varied across studies. Caregiver burden was most frequently assessed with the ZBI (*k* = 33), followed by the Neuropsychiatric Inventory–Distress scale (NPI‐D, *k* = 2). One study each used the Caregiver Burden Inventory (CBI), the CDI, and the SCB. Neuropsychiatric symptoms were assessed using the NPI‐12 in all studies (*k* = 15). Patient depression was evaluated with the NPI‐12 depression subscale (*k* = 5), the Cornell Scale for Depression in Dementia (CSDD) (*k* = 2). Quality of life was assessed in eight studies using the Quality of Life in Alzheimer's Disease (QoL‐AD) scale and in one study using the World Health Organization Quality of Life – Brief Version (WHOQOL‐BREF). Cognitive status (*k* = 9) was most frequently assessed with the Mini‐Mental State Examination (MMSE, *k* = 8), while one study used the Cambridge Cognitive Examination (CAMCOG). Functional status (*k* = 10) was measured using a range of instruments, including the Functional Activities Questionnaire (FAQ, *k* = 3), Functional Independence Measure (FIM, *k* = 1), Functional Disability Scale (FDS, *k* = 1), Edinburgh Feeding Evaluation in Dementia (EdFED, *k* = 1), Disability Assessment for Dementia (DAD, *k* = 2), Activities of Daily Living Questionnaire (ADL‐Q, *k* = 1), and the Social and Emotional Functioning scale (*k* = 1). Caregiver depression (*k* = 16) was most frequently evaluated with the Beck Depression Inventory (BDI, *k* = 6), followed by the Patient Health Questionnaire‐9 (PHQ‐9, *k* = 5). The remaining studies each used a single instrument, including the Depression Anxiety Stress Scales (DASS‐21, *k* = 1), the Geriatric Depression Scale (GDS, *k* = 1), the Center for Epidemiologic Studies Depression Scale (CES‐D, *k* = 1), and the Hospital Anxiety and Depression Scale (HADS, *k* = 1). Disease awareness (*k* = 6) was assessed with the ASPIDD in all studies. Caregiver anxiety (*k* = 6) was most frequently assessed with the Beck Anxiety Inventory (BAI, *k* = 3), while one study each used the Hamilton Anxiety Rating Scale (HAM‐A, also reported as HAI), the Depression Anxiety Stress Scales (DASS‐21), and the Hospital Anxiety and Depression Scale (HADS).

### 3.2 Quality assessment and risk of bias

Studies were independently assessed using the NOS^27^ across three domains: participant selection, group comparability, and outcome assessment. Discrepancies were resolved through discussion. Across the 40 included studies, scores ranged from 5 to 9 (M = 7.2, SD = 1.0). Based on standard thresholds, 30 studies (75.0%) were rated as high quality (≥7), 10 (25.0%) as moderate quality (5–6), and none as low quality (<5). At the item level, all studies met criteria for sample representativeness, outcome ascertainment, and adequacy of statistical testing. Adequate sample size was documented in 30 studies (75.0%), whereas non‐response was adequately addressed in 11 studies (27.5%). In the comparability domain, 24 studies (60.0%) achieved the maximum score, 14 (35.0%) showed partial comparability, and 2 (5.0%) did not meet this criterion. For outcome assessment, 23 studies (57.5%) achieved the maximum score and 17 (42.5%) a partial score. Overall, the main potential sources of bias were limited reporting on non‐respondents, variability in sample size adequacy, and inconsistent control of confounding factors. Table 1 presents the individual quality ratings.

**TABLE 1:** Newcastle–Ottawa scale for methodological quality

N	Study	Title	Representativeness of the sample*	Representative sample Sample size*	Non‐respondents*	(Demonstration of the outcome of interest ‐validated samples)*	Comparable design**	Assessment or outcome**	Statistical test*	Total score
1	Mangone et al^55^	Influence of feelings of burden on the caregiver's perception of the patient's functional status	*	‐	‐	*	**	*	*	6
2	Allegri et al^36^	Neuropsychiatric symptoms as a predictor of caregiver burden in Alzheimer's disease	*	*	‐	*	**	*	*	7
3	Moscoso et al^59^	Profile of caregivers of Alzheimer's disease patients attended at a reference center for cognitive disorders	*	‐	‐	*	*	**	*	6
4	Truzzi et al^70^	Burnout in a sample of Alzheimer's disease caregivers in Brazil	*	*	‐	*	**	**	*	8
5	Fialho et al^51^	Dementia caregiver burden in a Brazilian sample: Association to neuropsychiatric symptoms	*	*	*	*	*	*	*	7
6	Arango Lasprilla et al^38^	The effect of dementia patient's physical, cognitive, and emotional/behavioral problems on caregiver well‐being: Findings from a Spanish‐speaking sample from Colombia, South America	*	*	‐	*	*	**	*	7
7	Balieiro et al^40^	Caregiver distress associated with behavioral and psychological symptoms in mild Alzheimer's disease	*	*	*	*	*	*	*	7
8	Moreno et al^72^	Necesidades familiares y su relación con las características psicosociales que presentan los cuidadores de personas con demencia [Family needs and their relationship with psychosocial functioning in caregivers of people with dementia]	*	*	‐	*	*	**	*	7
9	Christofoletti et al^45^	Physical activity attenuates neuropsychiatric disturbances and caregiver burden in patients with dementia	*	‐	‐	*	**	**	*	7
10	Canonici et al^44^	Functional dependence and caregiver burden in Alzheimer's disease: A controlled trial on the benefits of motor intervention	*	‐	*	*	**	**	*	8
11	Slachevsky et al^16^	The CUIDEME Study: Determinants of Burden in Chilean Primary Caregivers of Patients with Dementia	*	*	*	*	**	**	*	9
12	Sutter et al^68^	Linking family dynamics and the mental health of Colombian Dementia Caregivers	*	*	‐	*	**	**	*	8
13	Rosas‐Carrasco et al^64^	Caregiver burden of Mexican dementia patients	*	*	‐	*	**	**	*	8
14	Perrin et al^15^	Connecting health‐related quality of life and mental health in dementia caregivers from Colombia, South America	*	*	*	*	‐	**	*	7
15	Corazza et al^46^	Los predictores psiconeuroinmunologicos de la sobrecarga de cuidado en ancianos cuidadores de pacientes con enfermedad de Alzheimer	*	‐	‐	*	*	**	*	6
16	Santos et al^65^	Caregivers’ quality of life in mild and moderate dementia	*	*	‐	*	**	**	*	8
17	Ramirez et al^63^	La carga de los cuidadores de adultos mayores con demencia en una región rural [The burden to caregivers of aged people suffering dementia in a rural zone]	*	‐	‐	*	*	*	*	5
18	Medrano et al^56^	Burden, anxiety and depression in caregivers of Alzheimer patients in the Dominican Republic	*	*	*	*	*	*	*	9
19	Moreno et al^58^	Caregiving in Dementia and its Impact on Psychological Functioning and Health‐Related Quality of Life: Findings from a Colombian Sample	*	*	‐	*	**	**	*	8
20	Storti et al^67^	Neuropsychiatric symptoms of the elderly with Alzheimer's disease and the family caregivers' distress	*	*	*	*	‐	*	*	6
21	Sousa et al^66^	Factors associated with caregiver burden: Comparative study between Brazilian and Spanish caregivers of patients with Alzheimer's disease (AD)	*	*	‐	*	**	**	*	8
22	Sutter et al^69^	Beyond Strain: Personal Strengths and Mental Health of Mexican and Argentinean Dementia Caregivers	*	*	‐	*	**	*	*	7
23	Paredes et al^61^	Structural equation model linking dementia cognitive functioning, caregiver mental health, burden, and quality of informal care in Argentina	*	*	‐	*	**	**	*	8
24	Amorim et al^14^	Social skills and well‐being among family caregivers to patients with Alzheimer's disease	*	‐	‐	*	**	*	*	6
25	Belfort et al^43^	The relationship between social cognition and awareness in Alzheimer disease	*	*	‐	*	*	*	*	6
26	Aravena et al^39^	Measuring change in perceived well‐being of family caregivers: Validation of the Spanish version of the Perceived Change Index (PCI‐S) in Chilean dementia caregivers	*	*	‐	*	*	**	*	7
27	Carletti Pessotti et al^62^	Family caregivers of elderly with dementia: Relationship between religiosity, resilience, quality of life and burden	*	*	‐	*	**	**	*	8
28	Kimura et al^52^	Caregivers’ perspectives of quality of life of people with young‐ and late‐onset Alzheimer disease	*	*	‐	*	**	**	*	8
29	Delfino et al^49^	Path analysis of caregiver characteristics and neuropsychiatric symptoms in Alzheimer's disease patients	*	*	‐	*	**	*	*	7
30	Baptista et al^41^	Differences in awareness of disease between young‐onset and late‐onset dementia	*	*	‐	*	**	**	*	8
31	Kimura et al^53^	Young‐ and late‐onset dementia: A comparative study of quality of life, burden, and depressive symptoms in caregivers	*	*	‐	*	**	**	*	8
32	Nogueira et al^60^	Quality of life of people with Alzheimer disease: Comparison between dyads degree of kinship	*	*	‐	*	**	*	*	7
33	Mora‐Castaneda et al^57^	Carga, depresión y familismo en cuidadores informales colombianos de pacientes con esquizofrenia y pacientes con demencia [Burden, depression and familism in Colombian informal caregivers of patients with schizophrenia and patients with dementia]	*	*	*	*	*	*	*	7
34	Delfino et al^50^	Neuropsychiatric symptoms associated with family caregiver burden and depression	*	‐	‐	*	**	**	*	7
35	Moreira et al^71^	Caregiver burden related to feeding process in Alzheimers disease	*	*	‐	*	*	*	*	6
36	De Araujo et al^48^	Psychosocial factors affected by burden in family caregivers of people with Alzheimer's disease	*	‐	‐	*	*	*	*	5
37	Silva‐Sauer et al^47^	Physical activity and its relationship to burden and health concerns in family caregivers of people with dementia	*	*	*	*	**	**	*	9
38	Lin et al^54^	Caregiver Burden among Caregivers of Older Adults with Alzheimer's Disease Impairs their Quality of Life: A Cross‐sectional Study in Brazil	*	‐	*	*	*	*	*	6
39	Barbosa et al^42^	Validation of the Pearlin Mastery Scale for unpaid caregivers of people living with dementia in Brazil: A methodological study.	*	*	*	*	**	*	*	8
40	Alvarez Polo et al^37^	Relationship between caregiver characteristics and the reported quality of life of people with mild and moderate dementia.	*	*	‐	*	**	**	*	8

Each asterisk (*) represents a score in the NewcastleOttawa Scale. More asterisks indicate higher methodological quality according to the scale. Studies were scored from 0 to 9, where scores ≥7 indicate high quality, scores of 56 indicate moderate quality, and scores <5 indicate low quality.

### 3.3 Meta‐analysis results

The number of studies contributing to each meta‐analysis (k) varied across variables depending on data availability.

#### 3.3.1 Associations with patient‐related factors

##### Patient neuropsychiatric symptoms and caregiver burden

**FIGURE 3:**
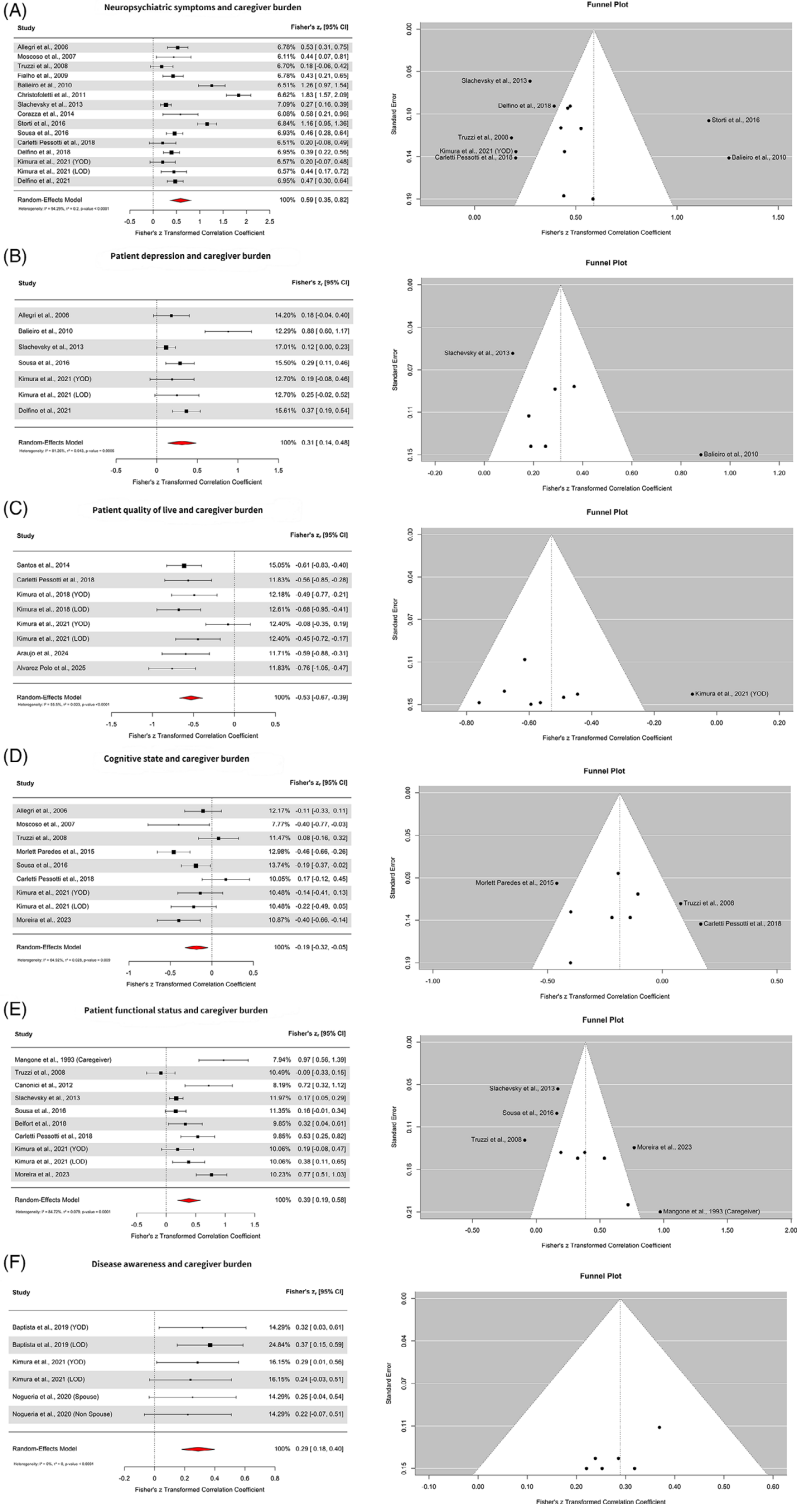
Forest plots and funnel plots of patient‐related factors associated with caregiver burden. (A) Neuropsychiatric symptoms: higher symptom severity was associated with greater caregiver burden. (B) Patient depression: higher depressive symptoms were associated with greater caregiver burden. (C) Patient quality of life: better quality of life was associated with lower caregiver burden. (D) Patient cognitive status: better cognitive functioning was associated with lower caregiver burden. (E) Patient functional status: greater dependence in activities of daily living was associated with higher caregiver burden. (F) Patient disease awareness: lower disease awareness was associated with higher caregiver burden. Funnel plots are shown alongside each forest plot to illustrate the assessment of publication bias. Some studies contributed more than one effect size (e.g., subgroup analyses), which explains discrepancies between the number of studies and the total number of effect sizes (*k*).

Fifteen studies (*k* = 15, *n* = 1347) examined this association. More severe patient neuropsychiatric symptoms were linked to greater caregiver burden (*z* = 0.59, standard error [SE] = 0.12, 95% confidence interval [CI] [0.35, 0.83], *p* < 0.0001) (Figure 3A). Heterogeneity was high (I^2^ = 94.3%), but there was no evidence of publication bias (estimate = 0.39, *p* = 0.64). A leave‐one‐out analysis identified one outlier^45^; its exclusion reduced the effect (*z* = 0.49, SE = 0.09, 95% CI [0.33, 0.67], *p* < 0.0001) but heterogeneity remained high (I^2^ = 88.31%). Meta‐regression showed no significant moderating effects of caregiver age, education, sex, or patient diagnosis.

##### Subdomain analyses

To examine whether specific neuropsychiatric symptoms were differentially associated with caregiver burden, we conducted separate meta‐analyses for each symptom assessed with the NPI.^16^, ^36^, ^40^, ^50^, ^66^ The analyzed symptoms included hallucinations, agitation, apathy, appetite changes, irritability, euphoria, and motor disturbances (*k* = 5, *n* = 685 for all domains). All symptoms showed significant positive associations with caregiver burden. However, high heterogeneity and evidence of publication bias were observed for all symptoms except agitation. Detailed results are presented in Table S1.

##### Patient depression and caregiver burden

Seven studies (*k* = 7, *n* = 795) showed that higher levels of patient depression were associated with higher caregiver burden (*z* = 0.31, SE = 0.089, 95% CI [0.14, 0.49], *p* = 0.0005) (Figure 3B). Heterogeneity was considerable (I^2^ = 81.26%). No publication bias was identified (estimate = −0.07, *p* = 0.19). Influence analysis identified one study^40^ (*r* = 0.71) with a notably high correlation; its removal reduced the pooled effect (*z* = 0.23, SE = 0.05, 95% CI [0.13, 0.32]) and decreased heterogeneity (I^2^ = 34.28%). Meta‐regression showed that samples with higher caregiver education (*k* = 5) had a stronger association between patient depression and caregiver burden (β = 0.03, SE = 0.02, *z* = 2.31, *p* = 0.02, 95% CI [0.0051, 0.06]). Influence analysis indicated that no individual study unduly affected the regression coefficients.

##### Patient quality of life and caregiver burden

Eight studies (*k* = 8, *n* = 457) showed that higher patient quality of life was associated with lower caregiver burden (*z* = –0.53, SE = 0.07, 95% CI [–0.67, –0.39], *p* < 0.0001) (Figure 3C). Heterogeneity was moderate (I^2^ = 55.5%). No publication bias was detected in the analysis (estimate = –0.7, *p* = 0.89). Leave‐one‐out analysis identified one influential effect size from the young‐onset AD subgroup,^53^ where the association was close to zero (*r* = −0.078). Removing this effect size strengthened the pooled association (*z* = −0.59, SE = 0.05, 95% CI [−0.69, −0.49], *p* < 0.0001) and eliminated heterogeneity (I^2^ = 0.0%). Meta‐regression analyses showed no significant moderating effects of caregiver age, education, sex, or patient diagnosis.

##### Patient cognitive status and caregiver burden

Nine studies (*k* = 9, *n* = 632) indicated that better patient cognition was associated with lower caregiver burden (*z* = –0.19, SE = 0.07, 95% CI [–0.32, –0.05], *p* = 0.009) (Figure 3D). Heterogeneity was substantial (I^2^ = 64.9%) and no publication bias was identified (estimate = −0.22, *p* = 0.92). Meta‐regression showed that the protective effect of better cognition on caregiver burden was stronger in samples with a higher proportion of female caregivers (*k* = 9, β = 3.56, SE = 0.84, *z* = 4.24, *p* = 0.0001, 95% CI [1.91, 5.21]). Influence analysis showed no evidence of influential studies affecting the regression coefficients. Caregiver education initially showed no effect on the association between patient cognitive status and caregiver burden (*k* = 7, β = –0.034, SE = 0.031, *z* = –1.08, *p* = 0.28). However, influence diagnostics identified one study^71^ as highly influential (Cook's distance = 1.21, DFFITS = –1.64). After removing this study, caregiver education emerged as a significant moderator (*k* = 6, β = –0.06, SE = 0.02, *z* = –2.71, *p* = 0.01), indicating that higher caregiver education was associated with lower burden relative to patient cognitive status.

##### Functional status and caregiver burden

Ten studies (*k* = 10, *n* = 810) revealed that greater patient dependence in activities of daily living was associated with higher caregiver burden (*z* = 0.39, SE = 0.10, 95% CI [0.19, 0.58], *p* < 0.001) (Figure 3E). Heterogeneity was high (I^2^ = 84.7%), and Egger's regression test suggested potential publication bias (estimate = 0.64, *p* = 0.07). Meta‐regression did not detect significant moderating effects of caregiver age, education, sex, or patient diagnosis. Influence analysis showed no indication that any individual study unduly affected the regression coefficients.

##### Patient disease awareness and caregiver burden

Six studies (*k* = 6, *n* = 340) showed that lower patient disease awareness was associated with higher caregiver burden (*z* = 0.29, SE = 0.06, 95% CI [0.18, 0.39], *p* < 0.0001) (Figure 3F). No heterogeneity (I^2^ = 0%), publication bias (estimate = 0.71, *p* = 0.43), or influential studies were detected. Meta‐regression showed no significant moderating effects of caregiver age, sex, education, or patient diagnosis. Influence analysis showed no indication that any individual study unduly affected the regression coefficients.

#### 3.3.2 Associations with caregiver‐related factors

##### Caregiver depression and burden

**FIGURE 4 alz71506-fig-0004:**
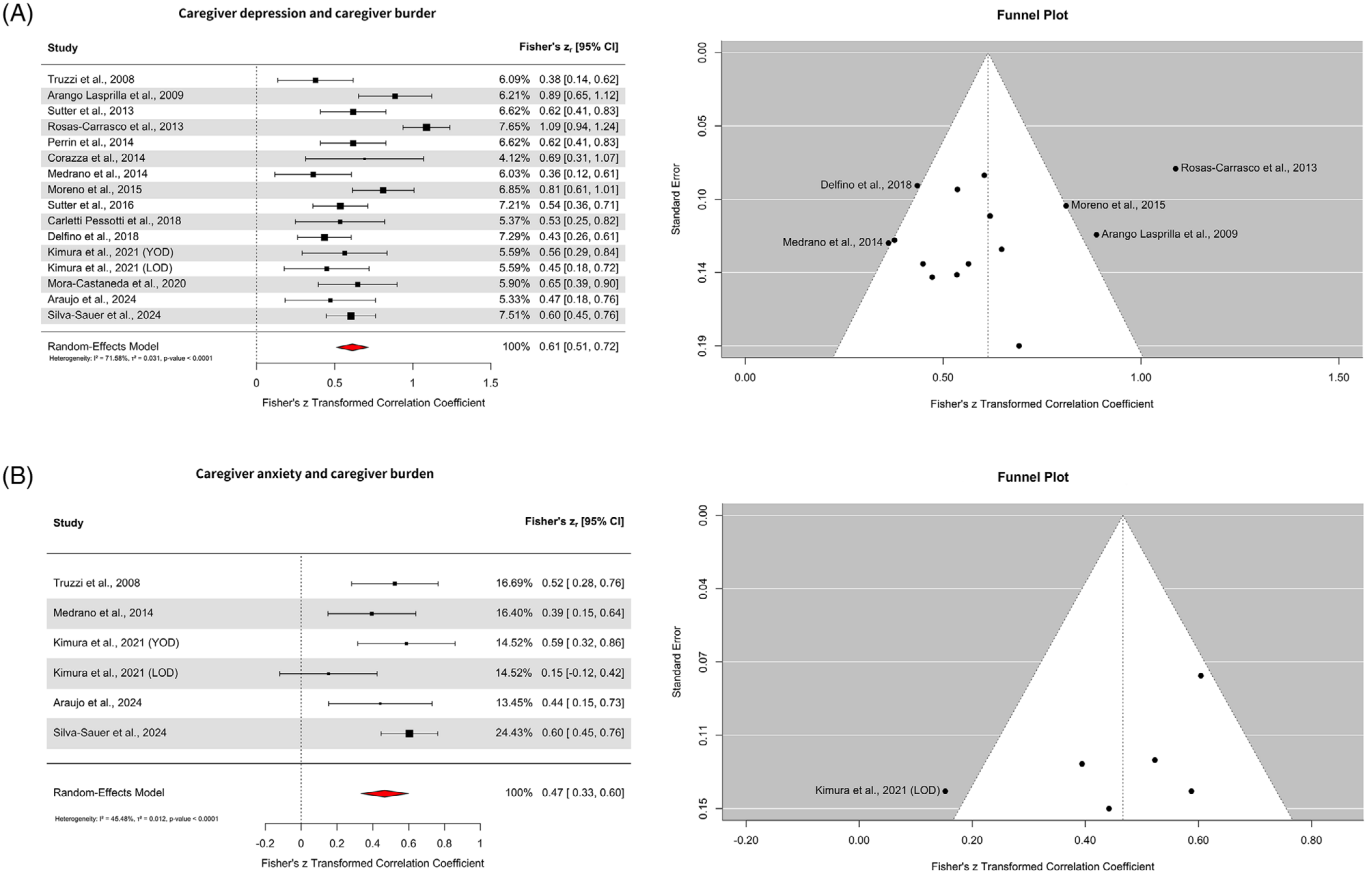
Forest plots and funnel plots of caregiver‐related factors associated with caregiver burden. (A) Caregiver depression: higher depressive symptoms were associated with greater caregiver burden. (B) Caregiver anxiety: higher anxiety symptoms were associated with greater caregiver burden. Funnel plots are shown alongside each forest plot to illustrate the assessment of publication bias.

Sixteen studies (*k* = 16, *n* = 1386) showed that greater depressive symptoms in caregivers were associated with higher caregiver burden (*z* = 0.61, SE = 0.05, 95% CI [0.51, 0.72], *p* < 0.0001) (Figure 4A). Heterogeneity was substantial (I^2^ = 71.6%) and no publication bias was found (estimate = 0.86 *p* = 0.24). Influence analysis identified one study^64^, which reported a comparatively large association (standardized coefficient = 0.80). Excluding this study slightly attenuated the pooled effect (*z* = 0.57, SE = 0.04, 95% CI [0.50, 0.65], *p* < 0.0001) and substantially reduced heterogeneity (I^2^ = 41.1%). Meta‐regression indicated a significant moderating effect of patient diagnosis (*k* = 15, β = 0.20, SE = 0.06, *z* = 3.28, *p* = 0.001), suggesting that the association between caregiver depressive symptoms and caregiver burden was stronger in studies including patients with AD and other dementias. No significant moderating effects were observed for caregiver age, sex, or education. Influence analysis showed no indication that any individual study unduly affected the regression coefficients.

##### Caregiver anxiety and burden

Six studies (*k* = 6; *n* = 452) showed that greater caregiver anxiety was associated with higher caregiver burden (*z* = 0.47, SE = 0.07, 95% CI [0.33, 0.60], *p* < 0.0001; Figure 4B). Between‐study heterogeneity was moderate (I^2^ = 45.48%). Egger's test showed no evidence of publication bias (intercept = 0.87, *p* = 0.16). Leave‐one‐out analysis identified one influential effect size^53^, which showed a comparatively small and non‐significant association with caregiver burden (standardized coefficient = 0.15). Excluding this effect size increased the pooled effect (*z* = 0.53, SE = 0.05, 95% CI [0.43, 0.63], *p* < 0.0001) and reduced heterogeneity to 0.0% (I^2^ = 0.0%). Meta‐regression showed no significant moderating effect of caregiver sex on the association between caregiver anxiety and caregiver burden (*k* = 5, β = 0.037, SE = 0.723, *z* = 0.05, *p* = 0.95). In the meta‐regression influence diagnostics, one study^47^ was identified as highly influential (Cook's distance = 1.98; DFFITS = 1.42); however, caregiver sex remained non‐significant after excluding this study (*k* = 4, β = −0.49, SE = 0.83, *z* = −0.59, *p* = 0.56). No significant moderating effects were observed for caregiver age, education, or patient diagnosis, and influence analyses indicated that no individual study unduly affected those regression coefficients.

## 4 DISCUSSION

This study presents the first systematic review and meta‐analysis examining factors associated with caregiver burden among informal dementia caregivers in LAC. Caregiver burden was most strongly associated with caregiver depression, and patient neuropsychiatric and depressive symptoms, while better patient quality of life and cognitive functioning were protective. Caregiver anxiety, patient functional dependence, and reduced disease awareness were also associated with higher burden, with smaller effect sizes. Higher caregiver education strengthened the association between patient depressive symptoms and caregiver burden, and female sex strengthened the association between patient cognitive status and caregiver burden. These findings underscore the need for interventions that jointly address patient behavioral and affective symptoms and caregiver mental health to reduce the impact of dementia caregiving in LAC^1^, ^13^.

The magnitude of the associations observed in this study is broadly consistent with prior meta‐analytic findings from the international literature, which has been predominantly derived from high‐income country settings.^17^, ^18^, ^19^, ^21^, ^22^ This convergence suggests that core determinants of caregiver burden are robust across contexts. However, in LAC these determinants operate in settings characterized by limited access to formal care services, high reliance on unpaid family caregiving, and greater socioeconomic inequality.^1^, ^3^, ^6^ As a result, similar levels of burden may translate into higher unmet needs and fewer resources to mitigate stress. By synthesizing evidence exclusively from LAC, this meta‐analysis provides a region‐specific characterization of caregiver burden and situates key correlates within this context, addressing a critical gap in the global literature. This is particularly relevant for informing context‐sensitive interventions and policy development in the region.

Neuropsychiatric symptoms exert a disproportionate influence by disrupting daily routines, heightening vigilance demands, and generating emotional uncertainty, which makes care more complex than physical dependence alone.^36^, ^40^, ^51^ These behavioral changes also increase the emotional demands of the caregiving relationship, and when patients experience agitation, apathy or irritability, caregivers often respond with heightened stress and reduced coping capacity.^16^, ^36^, ^50^ Depressive and anxiety symptoms in caregivers appear to play a central role in the experience of burden. While depressive symptoms reflect sustained emotional distress and exhaustion, anxiety may capture anticipatory stress, uncertainty, and heightened vigilance associated with managing unpredictable symptoms in dementia. These affective responses may co‐occur and reinforce each other, creating a dynamic that intensifies caregiver strain.^73^, ^74^ These effects are heightened in LAC due to limited psychological support, scarce behavioral management resources and the predominance of female informal caregivers,^3^, ^12^ who tend to experience higher levels of depressive and anxiety symptoms.^75^ Overall, the strong influence of behavioral and affective symptoms underscores how clinical demands interact with structural limitations in LAC to heighten caregiver burden.

Better patient cognition and quality of life were linked to lower caregiver burden, reflecting how preserved communication, engagement, and daily functioning ease emotional and practical demands on caregivers.^36^, ^50^, ^55^ When cognition declines, the loss of reciprocal understanding can heighten emotional distress and feelings of disconnection within the dyad.^51^ The relatively weak association between functional dependence and burden may be shaped by cultural norms in LAC, where assisting older relatives with daily tasks is often viewed as a moral obligation rather than a source of strain,^5^, ^12^ particularly when responsibilities are distributed across family members.^67^ These patterns underscore the importance of interventions that support cognitive stimulation and quality of life in people with dementia, while acknowledging the cultural values and shared caregiving practices that characterize the region.

Higher caregiver education may shape how depressive symptoms are recognized or reported, which could partly explain the stronger observed association with burden. The stronger associations observed in samples with a higher proportion of women are consistent with entrenched gendered caregiving roles in LAC, where emotional labor and expectations of self‐sacrifice may intensify strain.^1^, ^13^, ^58^, ^64^, ^76^ Likewise, greater effects in diagnostically mixed samples may reflect the added clinical heterogeneity and caregiving complexity associated with more variable symptom profiles and disease trajectories. Although most moderator effects were non‐significant, these findings should be understood within the broader structural context of LAC, where economic inequality, gender disparities, limited educational opportunities, and scarce formal care services constrain caregiver resilience and access to support.[Fig alz71506-fig-0004]

These findings support the Stress Process Model,^25^, ^26^ which conceptualizes caregiver burden as arising from sustained exposure to primary stressors associated with caregiving demands. In our study, neuropsychiatric and depressive symptoms emerged as central stressors, and caregiver depressive and anxiety symptoms may be understood as a parallel expression of psychological distress arising from prolonged stress exposure and closely intertwined with caregiver burden. The observed moderator effects further suggest that the impact of these stressors may vary according to caregivers’ educational, gendered, and diagnostic contexts. The Sociocultural Stress and Coping Model^77^ complements this interpretation by emphasizing that caregiving burden is shaped not only by care demands but also by culturally situated resources and appraisals that influence how stress is interpreted and managed. This perspective is especially relevant in LAC,^78^ where family‐centered care and gendered expectations may shape caregivers’ responses to dementia‐related demands.

This study offers several key strengths. It provides the first meta‐analytic synthesis focused exclusively on LAC, yielding region‐specific evidence that has been largely absent from the literature. The inclusion of diverse dementia subtypes and multiple caregiver and patient variables allows for a comprehensive and context‐sensitive assessment of burden. The use of meta‐regression helped identify potential sources of variability across studies, and strict adherence to PRISMA standards enhances methodological rigor and transparency.

Several limitations should be considered when interpreting these findings. The majority of studies were cross‐sectional, limiting inferences about temporal or causal relationships, and moderator effects should be interpreted cautiously given the relatively small number of studies per moderator, high heterogeneity and multiple comparisons. Although influence analyses identified some influential cases, heterogeneity often remained high after their exclusion, suggesting that variability reflected broader differences across studies rather than only isolated outliers. This heterogeneity also highlights the need for greater harmonization in the assessment and reporting of caregiver burden and related correlates across studies in LAC. Differences in study designs, measurement instruments, and reporting practices further complicate comparability across studies, and most available evidence originated from Brazil, reducing regional representativeness. Studies also relied on non‐probabilistic or convenience samples, which may have introduced sampling bias and limited the extent to which findings can be generalized to the broader population of dementia caregivers in LAC. Future studies should use more representative sampling strategies, including population‐based and multicenter designs, to improve generalizability across LAC. Potential small‐study effects were suggested only for functional status in the main analyses, whereas most exploratory analyses of individual neuropsychiatric symptom domains showed evidence of publication bias. Effects of patients’ diagnosis should be interpreted cautiously as a moderator, as it was coded using broad study‐level categories due to the lack of subtype‐specific results in most primary studies. Future studies should report findings separately by dementia subtype whenever possible to enable more precise analyses of diagnosis‐specific associations with caregiver burden. Contextual and structural determinants, including socioeconomic status, rurality and care policies, were rarely examined, underscoring major gaps in primary research rather than limitations of this meta‐analysis. Despite these constraints, this review adhered to PRISMA guidelines, applied a comprehensive search strategy and used a rigorous meta‐analytic framework, which strengthens confidence in the overall conclusions.

These findings underscore the need to integrate mental health screening and the systematic management of behavioral symptoms into routine dementia care in LAC, ensuring timely support for both caregivers and patients. Culturally adapted psychological interventions tailored to caregiver needs, together with cognitive stimulation programs may reduce burden and preserve quality of life. At the policy level, financial support, respite opportunities, and trained personnel to assist with patient care are essential components of equitable and sustainable care systems. Future research should adopt longitudinal and mixed‐method designs, incorporate structural determinants such as socioeconomic status, and rurality, and expand evidence from underrepresented countries to improve regional representativeness. Developing multilevel strategies that combine clinical, community, and policy approaches remains critical to reducing caregiver burden across the region.

In conclusion, this study provides the first comprehensive synthesis of factors associated with caregiver burden among informal dementia caregivers in LAC. The findings highlight the central role of behavioral and affective symptoms, as well as the protective contribution of patient cognition and quality of life. These insights emphasize the need for culturally grounded, gender‐sensitive, and multilevel interventions that integrate mental health support into dementia care. Strengthening caregiver support is essential for reducing burden and for advancing more equitable and sustainable dementia care systems across the region.

## CONFLICT OF INTEREST STATEMENT

Maria Alejandra Tangarife is employed full‐time by IGC Pharma SAS, a clinical‐stage pharmaceutical company. The other authors declare no competing interests. Author disclosures are available in the Supporting Information.

Marked Percentage: 3.3%

## Supporting information




Supporting Information

Supporting Information

Supporting Information
