# Supplementary material for: Factors linked to informal caregiver burden in dementia across Latin America and the Caribbean: A systematic review and meta‐analysis
Source: Alzheimers Dement. 2026 Jun 1;22(6):e71506. doi: 10.1002/alz.71506 (PMC13239666; doi:10.1002/alz.71506)
Supplement: Supplementary file 1 — Supporting Information [file ALZ-22-e71506-s001.docx]

**Supplementary Table 1.** Meta-analytic results for neuropsychiatric symptoms assessed with the Neuropsychiatric Inventory-12 (NPI-12)

| **Neuropsychiatric symptom**  **(k)** | **z** | **SE** | **95% CI** | **p** | **I²** | **Publication bias** | **Association with burden** |
| --- | --- | --- | --- | --- | --- | --- | --- |
| Hallucinations  (k =5) | 0.39 | 0.19 | 0.02, 0.77 | 0.037 | 95.37% | Yes  (p < 0.0001) | + |
| Agitation  (k = 5) | 0.29 | 0.05 | 0.201, 0.39 | <0.0001 | 26.52% | No  (p = 0.068) | + |
| Apathy  (k = 5) | 0.24 | 0.09 | 0.06, 0.42 | 0.009 | 79.18% | Yes  (p = 0.004) | + |
| Appetite  (k = 5) | 0.22 | 0.1 | 0.03, 0.41 | 0.02 | 81.91% | Yes  (p < 0.0001) | + |
| Irritability  (k =5) | 0.41 | 0.19 | 0.04, 0.78 | 0.03 | 95.38% | Yes  (p = 0.002) | + |
| Euphoria  (k =5) | 0.30 | 0.15 | 0.0009, 0.6 | 0.049 | 92.82% | Yes  (p = 0.0006) | + |
| Motor disturbances  (k =5) | 0.34 | 2.43 | 0.07, 0.61 | 0.015 | 91.41% | Yes  (p<0.0001) | + |

**Note.** All neuropsychiatric symptoms (NPS) were assessed using the Neuropsychiatric Inventory (NPI-12). Positive associations indicate higher caregiver burden with greater symptom severity.
