# Supplementary material for: Factors linked to informal caregiver burden in dementia across Latin America and the Caribbean: A systematic review and meta‐analysis
Source: Alzheimers Dement. 2026 Jun 1;22(6):e71506. doi: 10.1002/alz.71506 (PMC13239666; doi:10.1002/alz.71506)
Supplement: Supplementary file 2 — Supporting Information [file ALZ-22-e71506-s003.docx]

**Supplementary Table 2. Characteristics of studies included in the systematic review and meta-analysis**

| **Authors** | **Country** | **Sample** | **Participants’ Demographic characteristics** | | | **Clinical scales** | | **Main Results** |
| --- | --- | --- | --- | --- | --- | --- | --- | --- |
|  |  |  | **Mean age +/- SD or IQR** | **Sex**  **(female %)** | **Years of education**  **Mean +/- SD** | **Caregiver burden**  **Mean +/- SD**  **or**  **% Above Cut-off** | **Factors associated with caregiver burden**  **Mean +/- SD /**  **or % Above cut-off** |  |
| (Alvarez Polo et al., 2025) | Colombia | Informal caregivers of people with dementia  N = 50 | Caregivers:  Young: (27) 2.00 % (1)  Adult: (28–59) 50.00% (25)  Older adults: (≥60) 48.00% (24) | 90% | Studied years:  1 to 5: 6%  6 to 10: 14%  11 to 15: 52%  16 to 20: 26%  21 or more: 2% | ZBI:  No caregiver burden: 56%  Mild burden: 22%  Severe burden: 22% | Well-being  Medium 52.00%  High 48.00%  Uniqueness  Medium 52.00%  High 48.00%  Social interaction  Medium 66.00%  High 34.00%  Knowledge  Medium 4.00%  High 96.00%  Anticipation  Medium 16.00%  High 84.00%  Procedural/instrumental  Medium 6.00% High 94.00%  WHOQOL-BREF  Quality of life  Very poor 4.00% Poor 16.00%  Normal 24.00%  Good 48.00%  Very good 8.00% | Lower caregiver well-being and quality of life was associated with higher caregiver burden. Lower levels of caregiver uniqueness, social interaction, knowledge, and anticipation were also associated with higher burden. |
| (Barbosa et al., 2025)* | Brazil | Unpaid carers of people with dementia  N = 100  AD = 72%  Other types of dementia = 28% | 53.4 ± 9.32 | 95% | 90% (≥ 12 years of schooling) | ZBI: 62.85 ± 12.6 | Pearlin Mastery Scale  HADS (Hospital Anxiety and Depression Scale) - overall score  18.72 ± 7.74  HADS - depression  9.48 ± 3.77  HADS - anxiety  9.24 ± 4.31  QoL-AD – Overall score  30.54 ± 7.5 | Higher caregiver burden was associated with lower sense of mastery. |
| (Lin et al., 2025)* | Brazil | Caregivers of older adults with AD  N = 126 | 51.36 ± 10.90 | 96.7% | Incomplete / Complete Elementary School  7.9% (10)  Complete High School  31.7% (40)  Complete Higher Education  60.3% (76) | ZBI: 59.07 ± 15.83 | Caregiver Quality of Life (WHOQOL-Bref)  Physical domain  12.92 ± 3.39  Psychological domain  12.37 ± 3.27  Social relationships domain  11.89 ± 3.90  Environmental domain  12.73 ± 2.50  Self-assessment  12.50 ± 3.42 | Higher caregiver burden was associated with poorer caregiver QoL across domains, particularly in social relationships. |
| (Silva-Sauer et al., 2024) | Brazil | Family caregivers of people with dementia  N = 157  AD = 84.4%  Other types of dementia = NR | 46.4 ± 10.6 | 93% | Primary Education  31 ± 19.7  Secondary / Technical Education  67 ± 42.7  Higher Education  59 ± 37.6 | ZBI: 40.19 ± 19.9 | B-GBB-8, Giessen Subjective Complaints List Questionnaire -Portuguese Brazilian version – Overall  14.61 ± 7.9  HADS – overall  20.82 ± 8.7 | Burden was associated with somatic symptoms, but the strength of this relationship changed as a function of the physical activity level. |
| (de Araujo & Lacerda, 2024) | Brazil | Family caregivers of people with AD  N = 49 | 54.26 ± 8.99 | 100% | More than 8 years (77.6%) | 92% above the ZBI cut-off | Disease severity/staging (CDR): NR  Caregiver depression  (DASS-21 depression): Depression 65.31%  Caregiver anxiety  (DASS-21 anxiety): Anxiety 53.06%  Caregiver stress (DASS-21 stress): 55.10%  Quality of Life – Caregiver report of patient’s QoL (C-PQoL):  NR  Caregiver Mindfulness  (MASS): NR | Higher burden was significantly associated with more symptoms of depression, anxiety, and stress, and with lower QoL and mindfulness. |
| (Moreira et al., 2023) | Brazil | Primary/family caregivers of people with AD  N = 60  Patients:  AD = 60 | 57  (IQR 45–67) | 70% | 8 years  (median, IQR 6–12) | ZBI: 20.95 ± 6.5 | Feeding Functionality (EdFED):  3 (IQR 1.5–6)  Functional oral intake (FOIS): 7 (5-7)  Functional status (FAQ): 26 (IQR 18-30)  Care Time  5 (IQR 3–5)  Cognition (MMSE): 12 (IQR 6-15) | Greater burden was significantly associated with aversive feeding behaviors and longer caregiving time. These factors predicted caregiver distress even in early stages of the disease. |
| (Delfino et al., 2021) | Brazil | Family caregivers of people with AD  N = 134  Patients:  AD = 134 | 58.24 ± 12.6 | 80% | 14 ± 3.9 | ZBI: 31.46 ± 10.3 | Neuropsychiatric symptoms (NPI-12):  Apathy: 3.40 ±3.89  Anxiety: 2.76 ± 3.44  Dysphoria/depression: 2.12 ± 3.26  Delusions: 2.10 ± 3.26  Aberrant motor behavior: 2.87 ± 4.40  Irritability: 2.16 ± 3.38  Nighttime disturbances: 2.64 ± 4.08  Agitation/aggression: 1.96 ± 3.64  Appetite and eating disturbances: 2.20 ± 3.90  Disinhibition: 2.10 ± 3.79  Hallucinations: 1.37 ± 2.94  Euphoria/elation: 0.13 ± 0.88  Caregiver distress associated with Neuropsychiatric symptoms (NPI-D): 13 ± 9.07  Apathy-D: 1.61 ± 1.63  Anxiety-D: 1.40 ± 1.55  Dysphoria/depression-D: 1.21 ± 1.54  Delusions-D: 1.19 ± 1.56  Aberrant motor behavior-D: 1.13 ± 1.60  Irritability-D: 1.18 ± 1.66  Nighttime disturbances-D: 1.12 ± 1.62  Agitation/aggression-D: 0.91 ± 1.53  Appetite and eating disturbances-D: 0.93 ± 1.52  Disinhibition-D: 0.90 ± 1.49  Hallucinations-D: 0.79 ± 1.41  Euphoria/elation-D: 0.04 ± 0.36  Caregiver depression (BDI): 6.26 ± 5.98  Cognition (MMSE): 18 ± 5.9 | Most symptoms—especially agitation, depression, and delusions—were significantly associated with higher caregiver burden and depression levels. |
| (Kimura et al., 2021) | Brazil | Family caregivers of people with late (LOD) and young (YOD) onset of AD  N = 110  Patients:  Young-onset AD (mild) = 55  Late-onset AD (Severe) = 55 | 54.70 ± 14.5 | 85% | 12.03 ± 3.2 | ZBI: 27.48 ± 13.8 | Cognition (MMSE):  LOD: 18.56 ± 4.5  YOD: 15.27 ± 6.9  Functional Status (FAQ):  LOD: 16.95 ± 9.1  YOD: 20.42 ± 8.4  Quality of Life – Caregiver report of patient’s QoL (C-PQoL):  LOD: 31.89 ± 6.1  YOD: 28.13 ± 5.8  Quality of Life – Patient’s report (PQoL):  LOD: 33.91 ± 4.8  YOD: 34.02 ± 6.1  Quality of Life – Caregiver’s report (CQoL):  LOD: 38.62 ± 5.8  YOD: 37.6 ± 5.8  Caregiver Depression (BDI):  LOD: 5.60 ± 5.5  YOD: 7.95 ± 6.7  Caregiver Anxiety (BAI):  LOD: 4.49 ± 5.5  YOD: 7.29 ± 7.2  Patient depression (CSDD):  LOD: 7.22 ± 5.3  YOD: 9.78 ± 5.8  Neuropsychiatric symptoms (NPI-12):  LOD: 16.07 ± 14.8  YOD: 23.64 ± 19.7  Disease awareness (ASPIDD):  LOD: 9.44 ± 5.1  YOD: 9.22 ± 6.0 | Caregivers of individuals with young-onset dementia (YOD) reported significantly higher burden, depression, and hopelessness than those caring for LOD. Different factors predicted burden and mental health outcomes in each group, highlighting the distinct psychosocial challenges of YOD caregiving |
| (Nogueira et al., 2021) | Brazil | Informal caregiver’s spouses and non-spouses of people with AD  N = 98  Caregiver spouse  N = 49  Caregiver non-spouse  N = 49  Patients:  AD = 98 | (spouse-caregiver)  69.1 ± 10.1  (non-spouse caregiver)  50.8 ± 9.5 | (spouse-caregiver)  35%  (non-spouse caregiver)  42% | (spouse-caregiver)  10.3 ± 3.5  (non-spouse caregiver)  12.6 ± 2.4 | ZBI:  (spouse-caregiver)  33.3 ± 15.0  (non-spouse caregiver)  24.7 ± 13.5 | Spouse  MMSE  28.1 ± 1.2  Non-spouse  MMSE  29.0 ± 0.8  Spouse  BAI  6.6 ± 5.9  Non-spouse  BAI  7.0 ± 8.3  Spouse  BDI  8.7 ± 6.8  Non-spouse  BDI  6.7 ± 5.6  Spouse QoL  29.4 ± 5.9  Non-spouse QoL  31.1 ± 5.3 | In spouses, better QoL was linked to greater awareness of disease, while in non-spouses, it was linked to lower depression. Caregivers’ ratings were influenced by burden, mood, and their perception of the patient’s awareness |
| (Mora-Castañeda et al., 2020) | Colombia | Informal caregivers of people with dementia  N = 63 | 56.92 ± 9.91 | 90.5% | 39.7% with university education | ZBI  35.30 ± 15.23 | Hours of contact  71.4  Depression (CES-D):  18.22 ± 11.70  Familism (Familism Scale):  Family Obligation  3.83 ± 0.55  Social Support  3.65 ± 0.72 | Among Colombian caregivers, burden and depressive symptoms were higher in those caring for people with dementia. pa |
| (Baptista et al., 2019) | Brazil | Family caregivers of people with late (LOD) and young (YOD) onset of AD and vascular dementia  N = 132  Patients:  LOD -AD, n = 81  LOD-Vascular Dementia, n = 2  YOD-AD, n = 43  YOD-Vascular Dementia, n = 6 | PwLOD caregiver 58.66 ± 13.35  PwYOD caregiver 52.65 ± 15.52 | PwLOD caregiver 70%  PwYOD caregiver 84.3% | PwLOD caregiver 11.98 ± 2.86  PwYOD caregiver 13.09 ± 3.65 | ZBI:  PwLOD caregiver 29.60 ± 14.71  PwYOD caregiver 29.35 ± 13.58 | Awareness (ASPIDD)  LOD 10.48 ± 4.67  YOD: 8.45 ± 5.79  Cognition (MMSE):  LOD: 18.61 ± 4.16  YOD: 18.31 ± 4.19  Patient Depression (CSDD)  LOD: 7.42 ± 5.36  YOD: 9.53 ± 6.01  Neuropsychiatric Symptoms (NPI-12):  LOD: 17.46 ± 17.36  YOD: 18.12 ± 14.26  Quality of Life in AD – Patient report (PQoL-AD):  LOD: 34.49 ± 4.26  YOD: 33.45 ± 5.91  Quality of Life in AD – Caregiver report (CQoL-AD):  LOD: 36.81 ± 6.12  YOD: 37.43 ± 5.74  Quality of Life in AD – Caregiver report about patient QoL (QoL-AD):  LOD: 30 ± 5.94  YOD: 28.71 ± 5.88 | In both YOD and LOD, greater caregiver burden was associated with lower patient awareness and reduced QoL |
| (Aravena et al., 2018)* | Chile | Family caregivers of people with mild to moderate dementia  N = 94  Patients:  Mild Dementia = 59.6%  Moderate Dementia = 34%  Moderate – severe = 6.4% | 55.9 ± 14.14 | 86% | 10.3 ± 3.9 | ZBI-6:  8.5 ± 6.79 | Functional status (ADCS-ADL): 38.7±14.22  Dementia Stage  (GDS-R):  Mild: 59.6%  Moderate: 34.0%  Moderate – Severe: 6.4%  Neuropsychiatric Symptoms (NPI-Q):  21.8±14.03 (0-36)  Caregiver Health Perception (EQ-5D): 61.8 ± 23.98 (0-100) | Lower perceived well-being was significantly associated with greater caregiver burden, distress, behavioral symptoms in patients, and reduced patient functionality |
| (Pessotti et al., 2018) | Brazil | Family caregivers of people with AD and other dementias  N = 50  AD caregivers, n = 34  Other dementias caregivers, n = 16 | 54.7 ± 11.1 | 88% | 7.76 ± 3.9 | ZBI  33.6 ±17.3 | Caregiver perception of patient’s QoL (QoL-AD):  35.0 ± 5.7  Resilience scale (RS)  135.6 ± 22.5  Religiosity (DUREL):  Intrinsic religiosity: 3.10 ± 1.78  Organizational religiosity: 2.46 ± 1.45  Non-organizational religiosity: 4.0 ± 1.5  Cognitive Status:  MMSE  10.6 ± 8.1  Clock Drawing Test  1.8 ± 2.6  Neuropsychiatric Symptoms (NPI-10):  12.2 ± 10.2  Caregiver’s depression (BDI): 12.7 ± 11.1  Disability Assessment for Dementia (DAD): 37.4 ± 7.9 | Higher caregiver burden was associated with more neuropsychiatric symptoms in patients, lower caregiver QoL, and lower patient education. Greater resilience and intrinsic religiosity were linked to better caregiver well-being and less burden |
| (Delfino et al., 2018) | Brazil | Family caregivers of patients with AD.  N = 134  Patients:  AD = 134 | 58.24 ± 12.6 | 80% | 14 ± 3.9 | ZBI:  31.46 ± 10.3 | Cognition (MMSE): 18 ± 5.9    Dementia management Strategies Scale  29.69 ± 3.80  NPI-D  13 ± 9.07  BDI (caregiver depression)  6.26 ± 5.98 | Greater neuropsychiatric symptoms in patients were directly associated with caregiver burden and use of criticism-based management strategies. Caregiver burden also mediated the effects of depressive symptoms and active management on patient symptoms. Women reported more distress and symptoms overall. |
| (Kimura et al., 2018) | Brazil | Family caregivers of people with late (LOAD) and young (YOAD) onset of AD  Patients:  N = 110  LOD AD – n = 55  YOD AD – n = 55 | LOAD: 60.8 ± 13.0  YOAD:  54.0± 14.3  Total:  57.6 ± 14.0 | LOAD: 73.6%  YOAD: 52.8%  Total:  63.6% | LOAD: 9.7 ± 3.8  YOAD:  11.1 ± 3.1  Total:  10.4 ± 3.6 | ZBI:  LOAD: 27.0 ± 13.7  YOAD: 27.0 ± 15.0  Total:  NR | Dementia Severity mild, n (%) (CDR):  LOAD: 35 (61.4%)  YOAD: 38 (71.6%)  Cognition (MMSE):  LOAD: 20.6 ± 4.0  YOAD: 20.0 ± 3.5  Functional Status (FAQ):  LOAD: 15.3 ± 8.5  YOAD: 18.6 ± 6.9  Patient Depression (CSDD):  LOAD: 3.6 ± 2.5  YOAD: 6.2 ± 3.7  Quality of Life in AD – Patient report (PQoL-AD):  LOAD: 29.6 ± 7.8  YOAD: 29.4 ± 6.4  Quality of Life in AD – Caregiver report about patient QoL (QoL-AD):  LOAD: 32.9 ± 5.8  YOAD: 33.6 ± 6.5 | In both YOAD and LOAD, greater caregiver burden was significantly associated with lower caregiver-rated QoL of the person with dementia. |
| (Belfort et al., 2018) | Brazil | Family caregivers of people with AD  N = 50  Patients:  AD = 50 | 60.16 ± 13.86 | 82% | 11.84 ± 2.73 | ZBI:  28.36 ± 15.14 | Social and Emotional Functioning (SEQ):  0.05 ± 0.15  Disease Awareness (ASPIDD): 9.4 ± 4.8  Cognition:  MMSE:  Patient:19.22 ± 3.96  Caregiver: 28.98 ± 1.5  ADAS-Cog: 24.34 ± 8.50  DIGIT SPAN: 10.94 ± 3.81  Patient Depression (CSDD): 7.72 ± 5.10  Functional Status (pf-FAQ): 15.94 ± 8.29  QoL in AD – Patient report (PQoL-AD):  34.80 ± 4.20  Quality of Life in AD – Caregiver report (CQoL-AD):  36.98 ± 6.01  Quality of Life in AD – Caregiver report about patient QoL (QoL-AD):  30.72 ± 5.45  Neuropsychiatric Symptoms (NPI): 13.86 ± 12.71 | Lower social and emotional functioning (SEF) was significantly associated with poorer awareness of social relationships, reduced QoL, and higher caregiver burden. SEF was not related to cognitive performance |
| (Araujo de Amorim et al., 2017)* | Brazil | Family caregivers of AD patients  N = 41 | 61.9  (ranging from 40 to 80, and predominantly 50-60 years of age) | 87.8% | Complete High School  34.14%  Complete Higher Education  46.34% | ZBI: NR | Social Skills Inventory (SSI-Del-Prette: NR  WHO-QoL-bref: NR | Family caregivers who have more elaborate repertoire of social skills tend to have a better QoL and experience less burden |
| (Sousa et al., 2016) | Brazil | Family caregivers of people with AD  N = 128  Patients:  AD = 128 | 58.6 ± 13.6 | 78.1% | Schooling, n (%):  < 6 years = 7 (5.5 %)  ≥ 6 years = 121 (94.5%) | ZBI:  29.7 ± 17.7 | Functional Status:  DAD: NR  FAQ: NR  Basic Functional Status (ADL):  34.9 ± 5.5  Dementia Severity (CDR):  Mild: 70%  Moderate: 30%  Cognition:  MMSE:  19.1 ± 3.9  Neuropsychiatric Symptoms (NPI):  15.9 ± 14.8  Hallucinations: 0.3 ± 1.2  Delusions: 0.7 ± 1.9  Agitation/Aggression: 0.3 ± 1.2  Depression: 1.1 ± 2.7  Anxiety: 2.6 ± 3.5  Euphoria: 0.3 ± 1.1  Apathy: 2.8 ± 3.8  Disinhibition: 0.4 ± 1.4  Irritability: 1.8 ± 3.0  Motor disorders: 1.1 ± 2.8  Sleep disorders: 1.3 ± 2.7  Eating disorders: 1.0 ± 2.5 | In Brazil, caregiver burden was higher among women and when patients did not attend Day Care Centers; depression and anxiety were the symptoms most strongly associated with burden |
| (Storti et al., 2016) | Brazil | Family caregivers of people with AD and Mixed dementia. 68 caregivers took care of their parents and 62 were reportedly living with the person with dementia.  N = 96  Patients with AD, n = 54  Patients with mixed dementia, n = 42 | 56 ± 10.6 | 90.6% | "High level of education"  (Does not report years of education) | Neuropsychiatric Inventory – Distress scale  (NPI-D):  30 ± 23.8 | Neuropsychiatric symptoms (NPI-12):  14.4 ± 11.9 | Greater neuropsychiatric symptom severity in dementia patients was strongly associated with higher caregiver distress. |
| (Sutter et al., 2016) | Argentina and Mexico | Informal primary caregivers of family members providing active daily care with dementia.  N = 127 | 57.14 ± 13.01 | 77.22% | 14.07 ± 4.67 | ZBI: NR | Life Orientation Test–Revised (LOT-R for optimism): NR  Sense of Coherence Scale–Short Form (SOC): NR  Brief Resilience Scale (BRS for caregiver disposition and management of caregiving): NR  Patient Health Questionnaire–9 (PHQ-9): NR  Satisfaction with Life Scale (SWLS): NR  Resilience Scale for Adults (RSA) | The purpose of the current study was to examine the relationships between personal strengths (optimism, SOC, and resilience) and the mental health of dementia caregivers from Mexico and Argentina. These personal strengths explained 32% of the variance in caregiver depression, 39% in burden, and 50% in SWL. |
| (Paredes et al., 2015) | Argentina | Family caregivers of people with dementia  N = 102 | 57.81 ± 13.49 | 74.5% | 14.17 | ZBI: NR | Caregiver depression (PHQ-9): NR  Satisfaction with Life (SWLS): NR  Caregiver anxiety (GAD-7): NR  Exemplary Care Scale (ECS): NR  Cognition (MMSE): NR | Higher satisfaction with life was associated with lower caregiver burden and better mental health outcomes. |
| (Moreno et al., 2015)* | Colombia | Informal caregivers of people with dementia (not specified)  N = 102 | 58.4 ± 13.3 | 81.4% | 9.1 ± 5.0 | ZBI: NR | Caregiver depression (PHQ-9): NR  Satisfaction with Life (SWLS): NR  Caregiver health related QoL (SF-36): NR | Caregivers with fewer depressive symptoms and higher life satisfaction reported better health-related QoL (HRQOL), especially in vitality and general health |
| (Corazza et al., 2014) | Brazil | Caregivers of patients with AD diagnosis  N = 30 | 71.3 ± 9.3 | 80% | 8.1 years | ZBI:  23.4 ± 15.1 | Patient Physical Activity (Modified Baeke questionnaire for elders): NR  Functional independence (MIF): NR  American Alliance for Health, Physical  Education, Recreation and Dance (AAHPERD): NR  Caregiver stress (Lipp): NR  Geriatric Depression - Caregiver (GDS-D):  5.8 ± 4.3  Neuropsychiatric symptoms (NPI): 16.2 ± 12 | Caregiver burden among elderly caring for AD patients was significantly predicted by depressive symptoms in the caregiver and neuropsychiatric symptoms in the patient |
| (Medrano et al., 2014) | Dominican Republic | Primary caregivers, responsible for taking care of patients with AD  N = 67 | 61 years average  Over 50 years  52% | 84% | Elementary education 55%  Professionals  28% | ZBI:  36% | Hamilton Scale (anxiety):  19% (13)  Symptomatological profile and depression severity:  43%   - Mild: 72% - Moderate: 10% - Severe: 17% | It showed a significant positive correlation between burden, anxiety, and depression, meaning that higher emotional distress is linked to greater caregiver burden.  Overall, the study highlights the need to include caregiver mental health assessment and support programs in dementia care. |
| (Perrin et al., 2014) | Colombia | Family/primary caregivers of people with dementia (AD, vascular, mixed, Parkinson’s)  N = 90  AD = 82  Vascular Dementia = 2  Parkinson’s disease dementia = 2  Mixed Dementia = 2 | 54.12 ± 11.50 | 64.4% | 15.18 ± 4.69 | ZBI:  NR | Health-related QoL (HRQOL – SF-36):  NR  Satisfaction with life (SWLS): NR  Caregiver depression (PHQ-9): NR | Higher health-related quality of life (HRQOL)—specifically vitality and fewer physical limitations—was strongly associated with lower depression and greater life satisfaction. Multiple regressions revealed that vitality, physical role limitations, and pain were significant predictors of caregiver burden. |
| (Ramírez et al., 2014) | Mexico | Informal and family caregivers of people with dementia  N = 15  Patients: Dementia (not specified) = 15 | 55.6 ± 16.01 | 73.3% | 5 ± 1.25 | ZBI:  Women  31.36 ± 11.58  Men  28 ± 4.96 | Caregiver’s mental health (SQR-20): NR | Caregiver burden was moderately and significantly correlated with psychological morbidity. Burden was higher among caregivers with greater emotional distress, those living with the care recipient, and spouse caregivers |
| (Santos et al., 2014) | Brazil | Informal caregivers of people with mild to moderate AD.  N = 88  Patients:  AD = 88  Mild = 43  Moderate = 45 | 59.22 ± 13.83 | 76.1% | 11.43 ± 3.49 | ZBI:  30.48 ± 18.59 | CQoL  35.36 ± 7.35  Dementia severity (CDR): NR  Cognition (MMSE): 19.07 ± 4.41  Functional Status (FAQ): 16.74 ± 9.54  Patient depression (CSDD): 8.61 ± 5.19  Disease Awareness (ASPIDD): NR  Neuropsychiatric Symptoms (NPI-12): 16.31 ± 14.48  Caregiver depression (BDI): 8.21 ± 8.60  Caregiver anxiety (BAI): 10.07 ± 10.25  Quality of Life in AD – Patient report (PQoL-AD):  34.80 ± 4.20  Quality of Life in AD – Caregiver report (CQoL-AD):  31.57 ± 6.32  Quality of Life in AD – Caregiver report about patient QoL (QoL-AD):  27.90 ± 5.73 | Caregiver QoL was negatively associated with burden, depressive symptoms, and anxiety in both mild and moderate dementia. While these factors were common across stages, the predictors of burden and depression differed by dementia severity |
| (Slachevsky et al., 2013) | Chile | Informal caregivers of people with dementia  N = 291  Patients:  Type of dementia non specified  N = 291 | 60.1 ± 13.9 | 74.9% | 7.3 ± 3.7 | ZBI:  63.99 ± 16.26 | Caregiver health (GHQ-12):  3.64 ± 2.75  Family functioning (Apgar Score):  7.03 ± 2.71  Neuropsychiatric symptoms (NPI-Q):  13.07 ± 8.46  Functional Status (SV-ADLQ):  67.34% ± 22.19%  Dementia severity (GDS)  5.25 ± 1.05 | High levels of burden in informal caregivers may be explained by psychological distress, familial disfunction and with patient-related factors |
| (Sutter et al., 2014) | Colombia | Family and Informal caregivers of people with dementia  N = 90  Patients:  AD = 82  Vascular Dementia = 4  Parkinson’s Disease = 2  Mixed dementia = 2 | 54.12 ± 11.50 | 64.4% | Graduated primary school = 4.4%  Some high school = 5.6%  Graduated high school = 16.7%  Some trade or technological school = 6.7%  Graduated trade or technological school = 12.2%  Some college/university = 4.4%  Graduated college/university = 18.9%  Postgraduate education = 31.1 | ZBI:  NR | Caregiver depression (PHQ-9):  NR  Satisfaction with life (SWLS):  NR  Caregiver perceived stress (PSS):  NR  Family adaptability and cohesion (FACES-IV):  NR  Family Communication (FCS):  NR  Family Satisfaction (FSS):  NR  Family functioning (FAD-GF):  NR | Better family dynamics (such as empathy, flexibility, and communication) were significantly associated with lower depression, stress, and higher life satisfaction. However, their relationship with caregiver burden was weak. |
| (Rosas-Carrasco et al., 2014) | Mexico | Family caregivers of people with dementia (possible AD, possible vascular dementia, mixed  dementia, frontotemporal, Lewy bodies and  Parkinson’s-associated dementia)  N = 175  Patients:  AD = 62  Vascular Dementia = 54  Mixed Dementia = 45  Other (Frontotemporal, Lewy bodies, Parkinson’s associated dementia) = 14 | 57.0 ± 13.3 | 82.3% | 12.4 ± 5.2 | Screen for Caregiver Burden (SCB): 21.8 ± 16.1 | Dysexecutive Questionnaire (DEX): 27.3 ± 18.5 (0-77)  Cognition (MMSE): 17 ± 6.5 (2-30)  Geriatric depression - Patient (GDS-D): 5.0 ±3.3 (0-15)  Instrumental functional status (Lawton IADL): 5.1 ± 5.4 (0-16)  Basic functional status (Barthel ADL): 74.4 ± 29.1 (0-100)  Number of Neuropsychiatric symptoms (NPI-12): 6.2 ± 3.2 (0-12)  Sleep disturbances Inventory (SDI): 3.1 ± 1.8 (0-8)  Caregiver depression (BDI):  9.4 ± 7.4 (0-33)  Caregiver anxiety (BAI): 9.8 ± 8.5 (0-50) | Patient dysexecutive syndrome, sleep disorders, and education level was significantly associated with higher caregiver burden, as well as caregiver depression. Patient-related variables had a stronger impact on burden than caregiver characteristics |
| (Canonici et al., 2012) | Brazil | Informal caregivers of people with AD from the community  N = 32  Patients:  AD = 32 | 54.2 ± 11.7 | 97.2% | 9.8 ± 4 | Control  ZBI:  35.6 ± 14.9  Intervention ZBI:  32.3 ± 14.7 | Cognition (MMSE): 15.5 ± 6.0  Dementia severity (CDR): 1.5  Neuropsychiatric symptoms (NPI):  Control: 19.6 ± 14.3  Intervention: 18.3 ± 13.8  Neuropsychiatric symptoms – Caregiver distress (NPI-D):  Control: 19.6 ± 14.3  Intervention: 18.3 ± 13.8  Functional Status (FIM):  Control: 99.5 ± 18.0  Intervention: 109.6 ± 12.5 | A 6-month motor intervention in Brazilian AD patients reduced functional decline and significantly lowered caregiver burden compared to controls |
| (Christofoletti et al., 2011) | Brazil | Informal caregivers of people with dementia  N = 59  Patients:  N =59  AD = 39%  Vascular = 32.2%  Mixed = 28.8% | 51 | 64.4% | Caregivers: 1 year | Median and confidence intervals:  NPI-D:  AD – Low Physical Activity: 17 (0-45)  AD – High Physical Activity: 14 (0-30)  Vascular dementia – Low Physical Activity: 18 (0-41)  Vascular dementia – High Physical Activity: 20 (16-24)  Mixed dementia – Low Physical Activity: 15 (6-24)  Mixed dementia – High Physical Activity: 17 (0-31) | Median and confidence intervals:  Dementia severity (CDR):  AD – Low Physical Activity: 2.5 (1-3)  AD – High Physical Activity: 2.5 (1-3)  Vascular dementia – Low Physical Activity: 3 (1-3)  Vascular dementia – High Physical Activity: 2 (1-3)  Mixed dementia – Low Physical Activity: 3 (1-3)  Mixed dementia – High Physical Activity: 3(1-3)  Cognition (CAMCOG):  AD – Low Physical Activity: 36.0 (0-100)  AD – High Physical Activity:37.5 (0-83)  Vascular dementia – Low Physical Activity: 17 (0-73)  Vascular dementia – High Physical Activity: 35 (0-16)  Mixed dementia – Low Physical Activity: 43.5 (0-79)  Mixed dementia – High Physical Activity: 28 (26-86)  Neuropsychiatric symptoms (NPI):  AD – Low Physical Activity: 36 (0-102)  AD – High Physical Activity: 28.5 (0-67)  Vascular dementia – Low Physical Activity: 49 (0-98)  Vascular dementia – High Physical Activity: 44 (20-72)  Mixed dementia – Low Physical Activity: 35 (0-60)  Mixed dementia – High Physical Activity: 36 (2-74)  AD – Low Physical Activity: 32.0 (17-55)  AD – High Physical Activity: 18.0 (8-31)  Vascular dementia – Low Physical Activity: 55 (47-58)  Vascular dementia – High Physical Activity: 31 (16-36)  Mixed dementia – Low Physical Activity: 10 (5-36)  Mixed dementia – High Physical Activity: 25 (5-52)  Physical Activity – patient (MBQE):  AD – Low Physical Activity: 0.5 (0.1-0.6)  AD – High Physical Activity: 3.0 (1-7.4)  Vascular dementia – Low Physical Activity: 1.0 (0.3-7.4)  Vascular dementia – High Physical Activity: 5.0 (1.9-6.3)  Mixed dementia – Low Physical Activity: 0.5 (0.3-0.9)  Mixed dementia – High Physical Activity: 1.5 (1.2-2.0) | Compared to the control group, the caregivers of patients with vascular dementia who engaged in physical activity had a reduced burden |
| (Balieiro et al., 2010) | Brazil | Family caregivers of people with mild AD.  N = 50  Patients: Mild AD = 50 | 53.9 ± 13.1 | 90% | NR | Caregivers Distress Index (CDI) 11.5 ± 10.41 | Neuropsychiatric symptoms (NPI-12):  Total NPI: 19.64 ± 18.05  NPI-D:  Depression: 3.8 ± 1.58  Apathy: 2.9 ± 1.69  Anxiety: 2.7 ± 1.58  Appetite/Eating Disturbances: 2.0 ± 1.80  Aberrant Motor Behavior: 2.9 ± 1.69  Delusions: 3.8 ± 1.58  Irritability: 3.6 ± 1.09  Hallucinations: 2.7 ± 1.94  Euphoria: 1.8 ± 1.49  Agitation: 3.7 ± 1.7  Disinhibition: 2.1 ± 1.51  Sleep Disturbances: 2.2 ± 2.12 | Caregiver distress was strongly linked to the severity of behavioral symptoms; especially irritability, delusions, and motor disturbances |
| (Moreno et al., 2010)* | Colombia | Family caregivers of people with dementia.  N = 73 | 57.7 ± 13.5 | 82.2% | 9.3 ± 4.9 | (ZBI):  31.5% little or no burden  31.5% mild burden  27.4% moderate  9.6% severe | Emotional Support  3.2 ± 0.43  Economic Support  2.5 ± 0.56  Time to rest  1.6 ± 0.51  Help with shores: NR  Health (PHQ-9): NR  Psychologic Support (SF-36): NR | Higher burden and depression were significantly predicted by the need for better physical health. Psychological support and rest needs predicted life satisfaction and social support, emphasizing the psychosocial toll of caregiving. |
| (Arango Lasprilla et al., 2009) | Colombia | Informal caregivers of people with dementia.  N = 73 | 57.7 ± 13.5 | 82.2% | 9.3 ± 4.9 | ZBI  31% No or little burden  32% mild-to-moderate burden  27% moderate to severe burden  10% severe burden | Caregiver depression (PHQ-9): NR  Interpersonal Support (ISEL-12): NR  Satisfaction with Life (SWLS): NR | Greater caregiver burden was associated with more severe dementia (CDR), lower functional independence (Barthel Index), and higher caregiver depression (CES-D). |
| (Fialho et al., 2009) | Brazil | Family caregivers of people with dementia.  N = 83  Patients:  Dementia (not specified) = 83 | 55.6 ± 12.8 | 83.1% | 8.2 ± 4.4 | ZBI:  31.35 ± 16.01 | Neuropsychiatric symptoms (NPI):  26.88 ± 22.86 | Higher caregiver burden was significantly associated with greater neuropsychiatric symptom severity in patients. No differences in burden were found based on caregiver age or gender. |
| (Truzzi et al., 2008) | Brazil | Informal caregivers of people with AD  N = 69  Patients:  AD = 69 | Burnout (yes)  54.3 ± 17.4  Burnout (no)  57.1 ± 13.9 | 85.5% | Elementary School  40.6 ± 28  High School  29.0 ± 20  College  30.4 ± 21 | BI (Burden interview):  35 ± 16  Burnout (yes) 48.5 ± 7.8  Burnout (no) 34 ± 15.9  Despersonalization  6.3 ± 6.5  Emotional Exhaustion  23.5 ± 14.9  Personal Accomplishment  37.5 ± 10.5 | Caregiver depression (BDI):  9 ± 7.8  Caregiver anxiety (BAI):  6.5 ± 7.9  Cognition (MMSE):  12.5 ± 6.5  Neuropsychiatric symptoms (NPI):  20.5 ± 17.2  Functional Status (FAQ):  22.4 ± 8.2 | Burnout was significantly associated with caregiver burden. Emotional exhaustion was the most prevalent dimension and was strongly linked to depression, anxiety, and patient behavioral symptoms |
| (Moscoso et al., 2007) | Brazil | Family caregivers of patients with AD attending at a Reference  Center for Cognitive Disorders.  N = 31  Patients:  AD = 31 | Without burden  58.33 ± 10.6  With burden  59.71 ± 13.3 | NR | Without burden  7.50 ± 3.87  With burden  10.14 ± 8.11 | ZBI:  31.77 ± 16.95 | Neuropsychiatric symptoms (NPI):  34.97 ± 25.64  Cognition  CAMCOG:  52.44 ± 13.63  MMSE: 15.55 ± 5.39  Functional Status (PFFEFER/FAQ):  17.45 ± 8.45 | Higher burden was significantly associated with greater neuropsychiatric symptoms and lower cognitive function in patients. Sociodemographic factors were not significantly related to burden |
| (Allegri et al., 2006) | Argentina | Primary caregivers of people with AD.  N = 82  Patients:  AD (very mild to severe) = 82 | 59.6 ± 14.8 | 81.5% | 9.3 ± 3.3 | ZBI:  29.2 ± 18.0 | Cognition (MMSE): 18.3 ± 3.9  Dementia severity (CDR): 1.6 ± 0.8  Neuropsychiatric symptoms (NPI): 24.0 ± 20.2 | Higher burden was significantly predicted by behavioral symptoms especially hallucinations, motor disturbances, and sleep issues. No significant association was found with cognition, dementia severity, or negative symptoms like apathy or depression. Lower caregiver education was also linked to greater burden. |
| (Mangone et al., 1993) | Argentina | Family caregivers of people with AD  N = 25  AD outpatient = 25 | 54 ± 14.71 | 64% | 10.6 ± 3.60 | CIB- ZBI:  30.6 ± 14.47 | Cognition (MMSE):  15.5 ± 7.13  Dementia Severity (BDRS):  8.7 ± 4.21  Basic Functional Status (ADL):  2.15 ± 1.90  Instrumental Functional Status (IADL):  6 ± 2.40  Functional Status:  FDS:  36.4 ± 11.8  DAFS:  66.35 ± 25 | Greater feelings of loss and sadness were significantly associated with higher caregiver burden. Emotional reactions, more than cognitive decline, were the main predictors of distress |

| ***Note.*** *NR = Not reported; AD = Alzheimer’s disease; ZBI = Zarit Burden Interview; ZBI-6 = Zarit Burden Interview – 6-item version; NPI = Neuropsychiatric Inventory (Cummings et al., 1994); NPI-12 = Neuropsychiatric Inventory – 12-domain version; Quality of Life = QoL; NPI-Q = Neuropsychiatric Inventory – Questionnaire version; NPI-D = Neuropsychiatric Inventory – Distress Scale; MMSE = Mini-Mental State Examination; CDR = Clinical Dementia Rating; FAQ = Functional Activities Questionnaire; ADL = Activities of Daily Living; IADL = Instrumental Activities of Daily Living; CIRS = Cumulative Illness Rating Scale; PHQ-9 = Patient Health Questionnaire-9; BDI = Beck Depression Inventory; BAI = Beck Anxiety Inventory; CES-D = Center for Epidemiologic Studies Depression Scale; HADS-A / HADS-D = Hospital Anxiety and Depression Scale – Anxiety / Depression subscales; SWLS = Satisfaction With Life Scale; SF-36 = Short Form Health Survey (36 items); HRQoL = Health-Related Quality of Life; RS = Resilience Scale (Wagnild & Young, 1993); PDUREL = Duke University Religion Index (Portuguese version: organizational, non-organizational, intrinsic religiosity); SSQ = Social Support Questionnaire; ECS = Exemplary Care Scale; ASPIDD = Assessment Scale of Psychosocial Impact of the Diagnosis of Dementia; DAFS = Direct Assessment of Functional Status; FOIS = Functional Oral Intake Scale; EdFED = Edinburgh Feeding Evaluation in Dementia; GDS = Global Deterioration Scale; CSDD = Cornell Scale for Depression in Dementia; QoL-AD = Quality of Life in AD Scale; PQoL = Patient QoL (QoL-AD version); C-PQoL = Caregiver report of patient QoL; CQoL = Caregiver’s own QoL report; LOD / YOD = Late-/Young-Onset Dementia; LOAD / YOAD = Late-/Young-Onset AD; SEQ = Social and Emotional Functioning Questionnaire; PA = Physical Activity; FIM = Functional Independence Measure; FSS = Family Support Scale; PwD = Person with Dementia; CAMCOG = Cambridge Cognitive Examination; WHOQOL-BREF = World Health Organization's Quality of Life assessment;* B-GBB-8 = Giessen Subjective Complaints List Questionnaire -Portuguese Brazilian version*; LOT-R =* Life Orientation Test–Revised; SOC = Sense of Coherence Scale; BRS = Brief Resilience Scale; RSA = Resilience Scale for Adults. *Studies marked with an asterisk (*) were not included in the quantitative synthesis because the variables associated with caregiver burden were examined in only one study or reported using non-standardized coefficients. (Barbosa et al., 2025; Lin et al., 2025; Aravena et al., 2018; Araujo de Amormim et al., 2017; Moreno et al., 2010; Moreno et al., 2015).* |
| --- |
